# Heterologous Expression and Functional Analysis of Rice GLUTAMATE RECEPTOR-LIKE Family Indicates its Role in Glutamate Triggered Calcium Flux in Rice Roots

**DOI:** 10.1186/s12284-016-0081-x

**Published:** 2016-03-08

**Authors:** Jun Ni, Zhiming Yu, Guankui Du, Yanyan Zhang, Jemma L. Taylor, Chenjia Shen, Jing Xu, Xunyan Liu, Yifeng Wang, Yunrong Wu

**Affiliations:** College of Life and Environmental Sciences, Hangzhou Normal University, Hangzhou, 310018 China; Department of Biochemistry, Hainan Medical College, Haikou, 571199 China; School of Life Sciences, Gibbet Hill Campus, University of Warwick, Coventry, CV4 7AL UK; State Key Laboratory of Rice Biology, China National Rice Research Institute, Chinese Academy of Agricultural Sciences, Hangzhou, 311400 China; State Key Laboratory of Plant Physiology and Biochemistry, College of Life Science, Zhejiang University, Hangzhou, 310058 China

**Keywords:** Aequorin, Glu, GLR, Calcium, HEK cells, Rice

## Abstract

**Background:**

Tremendous progress has been made in understanding the functions of the GLUTAMATE RECEPTOR-LIKE (GLR) family in *Arabidopsis*. Still, the functions of OsGLRs in rice, especially the ion channel activities, are largely unknown.

**Results:**

Using the aequorin-based luminescence imaging system, we screened the specificity of amino acids involved in the induction of Ca^2+^ flux in rice roots. Of all the amino acids tested, glutamate (Glu) was the only one to trigger Ca^2+^ flux significantly in rice roots. Detailed analysis showed a dose response of Ca^2+^ increase to different concentrations of Glu. In addition, the Ca^2+^ spike response to Glu was rapid, within 20 s after the application. A desensitization assay and pharmacological tests showed that the Glu-triggered Ca^2+^ flux is mediated by OsGLRs. Whole genome analysis identified 13 *OsGLR* genes in rice, and these genes have various expression patterns in different tissues. Subcellular localization studies showed that all the OsGLRs examined are likely localized to the plasma membrane. Bacteria growth assays showed that at least OsGLR2.1 and OsGLR3.2 have the potential to mediate ion uptake in bacteria. Further analysis using Fura-2-based Ca^2+^ imaging revealed a Glu-triggered Ca^2+^ increase in OsGLR2.1-expressing human embryonic kidney (HEK) cells.

**Conclusions:**

Our work provides a molecular basis for investigating mechanisms of Glu-triggered Ca^2+^ flux in rice.

**Electronic supplementary material:**

The online version of this article (doi:10.1186/s12284-016-0081-x) contains supplementary material, which is available to authorized users.

## Background

Glutamate (Glu), as a fast excitatory neurotransmitter, has an important role in the animal nervous system (Watkins and Jane 2006). Glu activates the ionotropic glutamate receptors (iGluRs) to mediate the Na^+^, K^+^ and Ca^2+^ entry into cells, catalyzing the signaling across the neurosynaptic gaps (Dingledine et al. 1999). Since the discovery of *GLUTAMATE RECEPTOR-LIKE* (*GLR*) genes in plants (Lacombe et al. 2001; Lam et al. 1998), which are closely related to the mammalian *iGluRs*, more and more evidence has indicated the existence of similar signaling pathways analogous to those found in animals (Forde 2014).

*Arabidopsis* has a total of 20 *GLR* genes, and phylogenetic analysis has shown that these genes can be subdivided into three clades (Chiu et al. 1999; Chiu et al. 2002; Lacombe et al. 2001). The basic domain structures of plant GLRs and animal iGluRs are conserved and phylogenetic analysis suggested that the separation of GLRs can be traced back to the last universal common ancestor of plants, metazoans and bacteria (Price et al. 2012). Recently, molecular genetics and electrophysiological approaches have made exciting advances in our understanding of various GLR functions in plants. AtGLR1.1 integrates and regulates the different aspects of carbon, nitrogen and water balance that are required for normal plant growth and development (Kang et al. 2004; Kang and Turano 2003). AtGLR1.2 mediates a plant signaling mechanism between male gametophytes and pistil tissue that is important for the pollen tube growth (Michard et al. 2011). AtGLR1.4 accounts for methionine (Met)-induced membrane depolarization in *Arabidopsis* leaves (Tapken et al. 2013). AtGLR3.3 is involved in plant defense signaling and the control of root gravitropism (Manzoor et al. 2013; Miller et al. 2010). AtGLR3.5 modulates cytosolic Ca^2+^ level to counteract effect of abscisic acid in seed germination (Kong et al. 2015). AtGLRs also mediate mechanical wound signaling and elicitor/pathogen-mediated defense signaling in *Arabidopsis* (Manzoor et al. 2013; Mousavi et al. 2013). Moreover, AtGLR3.2 and AtGLR3.4 were reported to form heteromeric channels to affect lateral root development via Ca^2+^ signaling in the phloem (Vincill et al. 2013).

In contrast with Glu or glycine (Gly) activated iGluRs, *Arabidopsis* GLRs have similar amino acid-gated ion channel activities, but with a broader agonist profile (Forde 2014). Six amino acids are considered to be the agonists of AtGLR3.3 dependent Ca^2+^ influx in *Arabidopsis*, and a mutation of *AtGLR3.3* gene inhibits the rise of Ca^2+^ triggered by these amino acids (Qi et al. 2006). Further research demonstrates that these six effective amino acids are not equivalent agonists, but grouped into hierarchical classes based on their ability to desensitize the response mechanism (Stephens et al. 2008). Human embryonic kidney (HEK) cells expressing AtGLR3.4 showed wide agonist profile with asparagine (Asn) and serine (Ser) as strong agonists and Gly less so (Vincill et al. 2012). Interestingly, AtGLR1.4 expressing in *Xenopus* oocytes, functioned as a cation channel that responded to an even broader range of amino acids with Met being the most effective and most potent agonist (Tapken et al. 2013). An aequorin-based luminescence recording system showed that strong Ca^2+^ responses were induced by all the amino acids tested, with the highest Ca^2+^ amplitude for cysteine (Cys) and lowest one for Asn (Zhu et al. 2013).

Compared with the great progresses of GLRs in dicotyledonous *Arabidopsis*, less information has been obtained from the monocot, rice. The only gene characterized was *OsGLR3.1*, which positively regulates cell proliferation in the root apical meristem and is required for survival of meristematic cells (Li et al. 2006). However, the ion channel activities of OsGLRs are still completely unknown.

In our previous research, we established an aequorin-based luminescence imaging system in rice, which was able to reflect the concentration of cytosolic free Ca^2+^ ([Ca^2+^]_i_) level in rice roots (Zhang et al. 2015). Here, using this system, we present the Glu-specific triggered Ca^2+^ response in rice roots. Further analysis using Fura-2-based Ca^2+^ imaging revealed a Glu triggered Ca^2+^ increase in OsGLR2.1-expressing HEK cells. Our results indicated an OsGLR mediated Ca^2+^ changes in response to Glu in rice roots.

## Results

### Glu Specifically Triggered Ca^2+^ Influx in Rice Roots

**Fig. 1:**
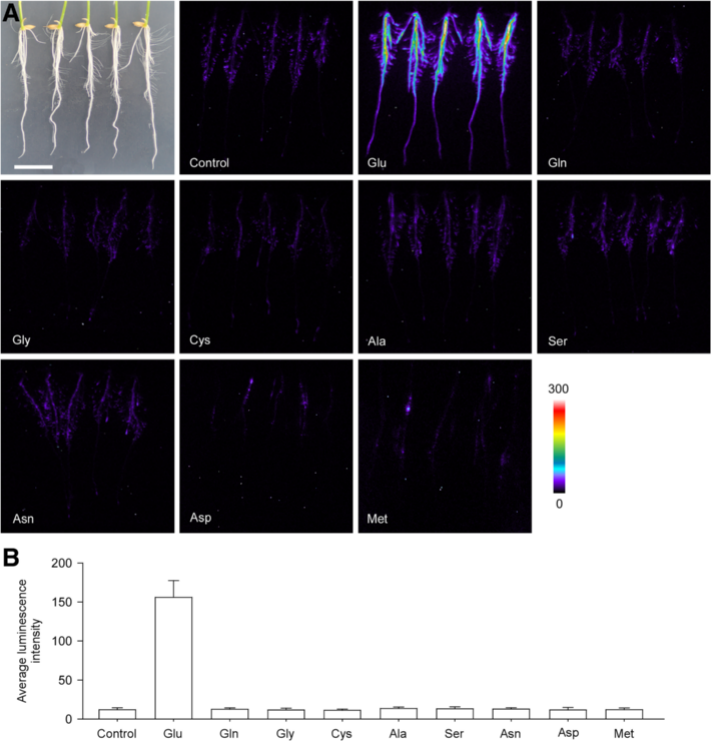
Glutamate specifically triggers Ca^2+^ influx in rice roots. **a** Pseudocolor images of aequorin luminescence in roots treated with different amino acids. The relationship between luminescence intensity and the pseudocolor images are scaled by a pseudocolor bar and the numbers next to the pseudocolor bar are maximum and minimum values of luminescence intensity. The image on the left of the top is the treated roots view in white light. Bar = 2 cm. Water was used instead of amino acid in the control treatment. **b** The bar chart of luminescence signal intensity of every treatment. The results were obtained from at least three independent experiments (mean ± sd; *n* = 10)

To investigate the amino acid-triggered Ca^2+^ influx in rice, we employed an aequorin-based luminescence imaging system, which is able to reflect the [Ca^2+^]_i_ level in rice roots (Zhang et al. 2015). After the reconstitution of aequorin by spraying seedlings with coelenterazine, the aequorin luminescence of rice roots was recorded using a photo-counting camera by treating plants with nine different amino acids respectively. As shown in Fig. 1, Glu induced a very strong [Ca^2+^]_i_ increase, while the other eight amino acids had little effect on the [Ca^2+^]_i_ induction compared with the control treatment. This showed that Glu is able to trigger Ca^2+^ influx specifically in rice roots.

### The Characteristics of [Ca^2+^]_i_ Response to Glu in Rice Roots

**Fig. 2:**
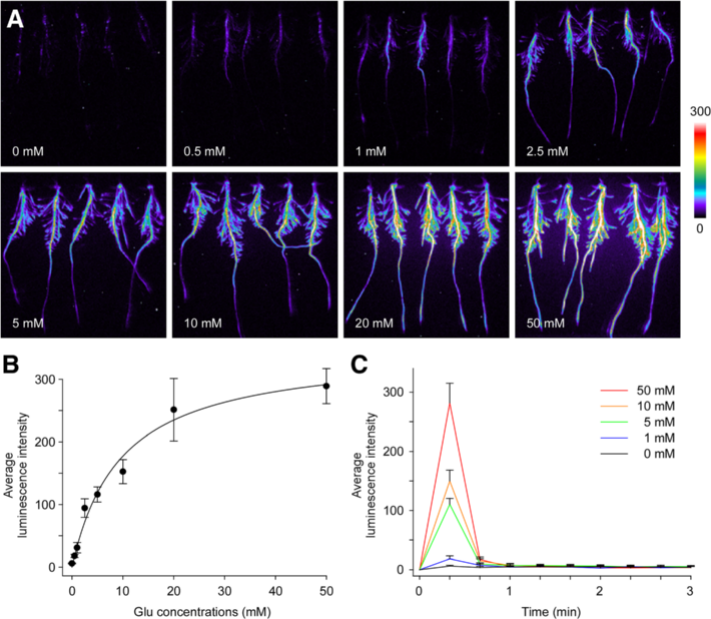
The characteristics of [Ca^2+^]_i_ response to Glu in rice roots. **a** Pseudocolor images of aequorin luminescence in roots treated with different concentrations of Glu. The relationship between luminescence intensity and the pseudocolor images are scaled by a pseudocolor bar and the numbers next to the pseudocolor bar are maximum and minimum values of luminescence intensity. **b** Concentration dependence of [Ca^2+^]_i_ increase in rice roots. Data were fitted by the Michaelis-Menten function. **c** The time courses of [Ca^2+^]_i_ changes induced by different concentrations of Glu in rice roots. The results were obtained from at least three independent experiments (mean ± sd; *n* = 10)

In order to investigate the [Ca^2+^]_i_ increase in response to Glu in rice roots, we examined the aequorin-based luminescence under various concentrations of Glu treatments. Overall, the more Glu that was applied, the stronger the luminescence signals would be (Fig. 2a). Detailed analysis showed that the concentration-dependent [Ca^2+^]_i_ increase was in agreement with Michaelis-Menten function with the Km value of 9.60 mM (Fig. 2b). To investigate the time courses of [Ca^2+^]_i_ responses induced by different concentrations of Glu in rice roots, the average luminescence intensity of continuous images with exposure time of 20 s, were analyzed and a comparison of basic parameters (amplitudes, durations and phases) of [Ca^2+^]_i_ responses were made. As shown in Fig. 2c, a strong luminescence signal was detected in the first image, which collected the luminescence signal within the first 20 s. However, almost no luminescence signal was detected after the first 20 s. This showed that Glu rapidly induced a sharp spike of [Ca^2+^]_i_ within 20 s, which quickly declined to the basal level after the spike. The amplitudes of luminescence signals varied according to different concentrations of Glu, while both the durations and phases were the same. These results were similar to the NaCl triggered [Ca^2+^]_i_ increase in rice and osmotic triggered [Ca^2+^]_i_ increase in *Arabidopsis* (Yuan et al. 2014; Zhang et al. 2015), indicating the existence of sensory channels to mediate Glu-triggered [Ca^2+^]_i_ increase in rice roots.

### Glu-Triggered [Ca^2+^]_i_ Increase is Mediated by OsGLRs

It is believed that GLRs are key components of a Ca^2+^ influx mechanism in response to amino acids in plants (Dennison and Spalding 2000). Thus, we examined whether the Glu-triggered [Ca^2+^]_i_ increase in rice roots is also mediated by OsGLRs. Desensitization is an important mode of ligand-gated Ca^2+^ channels at synapses in the animal nervous system (Jones and Westbrook 1996). Research in *Arabidopsis* showed that the phenomenon of desensitization also exists in the Glu activated, GLR mediated Ca^2+^ entry (Meyerhoff et al. 2005; Qi et al. 2006). To determine whether the Glu- triggered [Ca^2+^]_i_ increase is the manifestation of the same mechanism (e.g. the activation of OsGLRs), we examined the [Ca^2+^]_i_ increases in response to two sequential Glu treatments. The first application of Glu triggered an obvious [Ca^2+^]_i_ increase, while after one hour’s recovery, a second application of Glu with the same concentration, caused about 70 % reduction in the [Ca^2+^]_i_ increase compared with the first one (Additional file 1). To determine the dependence of *de novo* protein synthesis in the recovery of desensitization, we pretreated rice roots with cycloheximide (CHX), a well-known translation inhibitor. As a result, we observed a more serious inhibition (about 90 % reduction) in the [Ca^2+^]_i_ increase in response to a second application of Glu (Additional file 1). These results were very similar to that of AtGLR3.4-expressing mesophyll cells (Meyerhoff et al. 2005), indicating the possibility that OsGLRs mediate the Glu-triggered [Ca^2+^]_i_ increase in rice roots.

**Fig. 3:**
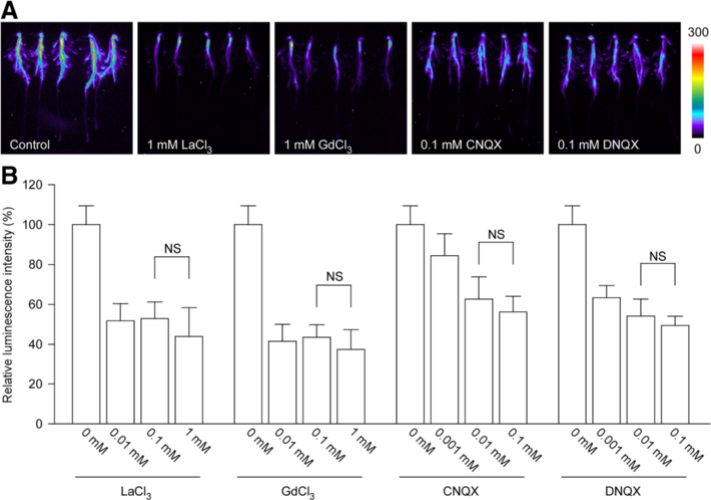
The pharmacological test of Glu-induced [Ca^2+^]_i_ increases. **a** Treatment of Ca^2+^ agonists (LaCl_3_ and GdCl_3_) and GLR-specific antagonists (CNQX and DNQX) reduced Glu-induced [Ca^2+^]_i_ increases. The relationship between luminescence intensity and the pseudocolor images are scaled by a pseudocolor bar and the numbers next to the pseudocolor bar are maximum and minimum values of luminescence intensity. **b** The effects of different concentrations of Ca^2+^ agonists and GLR-specific antagonists in Glu-induced [Ca^2+^]_i_ increases. The results were obtained from at least three independent experiments (mean ± sd; *n* = 10; NS, not significant *P* > 0.05; Student’s *t*-test)

To confirm the participation of OsGLRs in the Glu-triggered [Ca^2+^]_i_ increase, we conducted pharmacological tests in the aequorin-based luminescence imaging system. La^3+^ and Gd^3+^ are agonists of Ca^2+^, and they have been used as Ca^2+^ channel blockers to inhibit Ca^2+^ flux (Tracy et al. 2008). In our experiment, both LaCl_3_ and GdCl_3_ had significant inhibitory effects in Glu-triggered [Ca^2+^]_i_ increase. Furthermore, all the concentrations tested had similar inhibitory effects, indicating an effective inhibition function of LaCl_3_ and GdCl_3_ treatments (Fig. 3). 6-Cyano-7-Nitroquinoxaline-2,3-dione (CNQX) and 6,7-Dinitroquinoxaline-2,3-dione (DNQX) are GLR antagonists, and application of these drugs inhibits the channel activities of GLRs in plants (Meyerhoff et al. 2005; Michard et al. 2011; Tapken et al. 2013). In our experiment, both antagonists had significant inhibitory effects. In addition, dosage effects were observed in the inhibition of Glu-triggered [Ca^2+^]_i_ increase (Fig. 3). CNQX and DNQX were dissolved by Dimethyl sulfoxide (DMSO) in the stock solutions, and the highest concentration of DMSO in the working solution was 0.5 % (*v/v*). In order to investigate the effect of DMSO in the Glu-induced [Ca^2+^]_i_ increase, we examined the luminescence after various concentrations of DMSO pretreatments, and found that the effect 0.5 % of DMSO had no significant differences to the water control (Additional file 2). These results above showed that Glu-triggered [Ca^2+^]_i_ increase in rice roots is likely to be mediated by OsGLRs.

### Characterization of *OsGLR* Gene Family in Rice

**Fig. 4:**
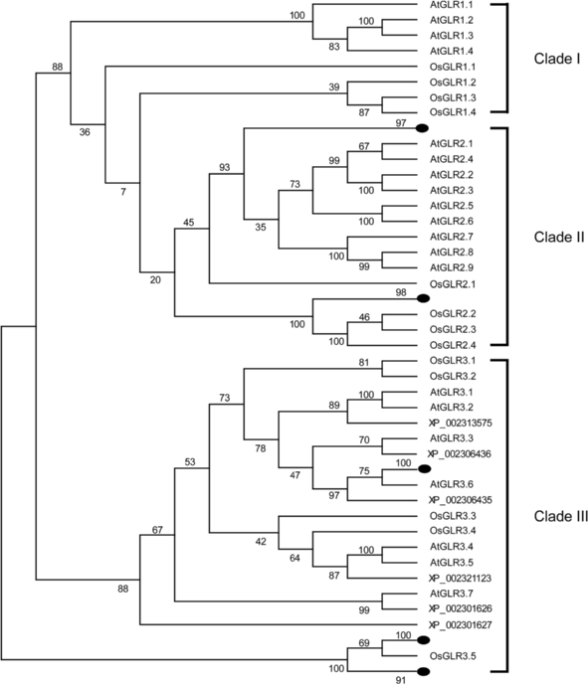
Phylogenetic relationships of GLRs in *Arabidopsis*, rice and poplar. Unrooted phylogenetic tree was constructed using MEGA6 by Maximum-Likelihood method. Three different GLR clades were marked by *lines. Black dots* represent compressed subtree of GLRs from poplar. [GenBank: XP_002313575, GenBank: XP_002306436, GenBank: XP_002306435, GenBank: XP_002321123, GenBank: XP_002301626 and GenBank: XP_002301627] are accession numbers of GLRs from poplar. Bootstrap values from 500 replicates are indicated at each branch

Since there is no comprehensive information about rice OsGLR family, searches of public genomic data were carried out. A total of 13 *OsGLR* genes that are most closely related to *Arabidospis* GLRs were identified by phytozome (http://www.phytozome.net/). Because there was no standard annotation assigned to these newly-identified genes, we named these genes according to their position in the phylogenetic tree (Fig. 4). The names of the *OsGLR* genes, the locus ID, the chromosome location, the isoelectric point (pI), the molecular weight (Mw) and predicted subcellular localizations are shown in Additional file 3. The decoded polypeptides were predicted to contain 3–6 transmembrane helices (TMHs), and most of them have the signature domains of animal iGluRs, including the ‘three-plus-one’ transmembrane domains (Lam et al. 1998) (Additional file 4). To detect the evolutionary relationships of GLRs, a phylogenetic tree was generated from alignments of 13 OsGLRs in rice and 20 AtGLRs in *Arabidopsis* (Fig. 4 and Additional file 5). We grouped the GLRs into three clades according to the previously reported work in *Arabidopsis* (Chiu et al. 2002). Detailed analysis showed that the phylogenetic relationships between *Arabidopsis* and rice are different among the three clades of GLRs. GLRs in clade I and clade II separated very early, while GLRs in clade III are evolutionary very close to each other (Fig. 4).

### Tissue-Specific Expression of *OsGLR* Genes

**Fig. 5:**
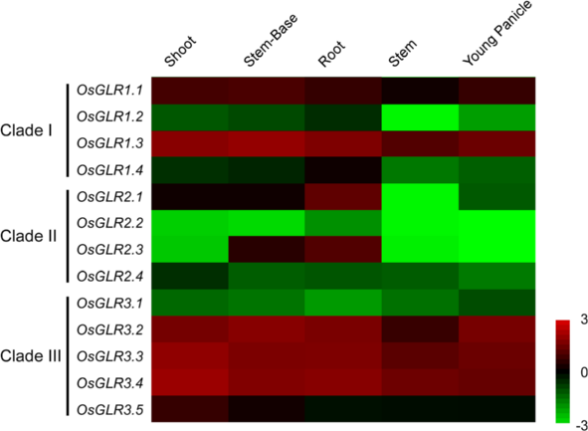
Tissue-specific expression patterns of *OsGLR* genes. The expression patterns of 13 *OsGLR* genes in five different tissues were analyzed. The name of *OsGLR* genes are indicated on the left of each panel, and different tissues are indicated on the top of the panel. The different colors correspond to the log-transcription values of the gene change-fold ratio shown in the bar at the right

We examined the expression patterns of identified *OsGLR* genes in the five different tissues by real-time quantitative reverse transcription polymerase chain reaction (qRT-PCR). The results revealed variations in the expression of *OsGLRs* in different tissues. In general, a number of *OsGLRs* were expressed at higher levels in all the tissues examined, compared with some members with overall lower levels. Interestingly, there were some *OsGLRs* with tissue-specific expression patterns with the highest expression levels seen in roots (Fig. 5). In clade I, high transcript levels of *OsGLR1.1* and *OsGLR1.3* were observed in all the tissues, in contrast with *OsGLR1.2* and *OsGLR1.4*, which had relatively higher expression in roots. In clade II, *OsGLR2.2* and *OsGLR2.4* had relatively low transcript levels in all the tissues, while *OsGLR2.1* and *OsGLR2.3* had very obvious tissue-specific expression patterns. *OsGLR2.1* was expressed most highly in the root, with moderate expression in shoot, stem-base and young panicle tissues, and almost no expression in the stem. *OsGLR2.3* had high expression in both root and stem-base tissues, and almost no expression in the other tissues. For clade III, all the genes except *OsGLR3.1*, had very high expression levels in all the tissues examined (Fig. 5).

We also examined the transcript level of *OsGLRs* after treatment of Glu and CNQX. We found that neither Glu nor CNQX treatment altered the transcript level of *OsGLRs* (Additional file 6). This indicated that although Glu and CNQX regulate the OsGLR mediated [Ca^2+^]_i_ increase in rice roots, they do not affect the transcript level of *OsGLRs*.

### OsGLRs Mediate ion Uptake in Bacteria

In order to investigate the functions of OsGLRs, we amplified the coding sequences (CDS) of *OsGLR1.1*, *OsGLR1.3*, *OsGLR2.1*, *OsGLR2.3*, *OsGLR3.2*, *OsGLR3.3* and *OsGLR3.4*, which have higher expression levels according to the previous qRT-PCR results. Interestingly, we found that *E. coli* transfected with plasmids inserted with *OsGLR2.1*, *OsGLR2.3*, *OsGLR3.2* had a slower growth rate than the others (Additional file 7A). To further investigate the ion transport functions of OsGLRs in bacteria, plasmids with *OsGLRs* were transfected into the K^+^ uptake-deficient *E. coli* strain LB650 (Stumpe and Bakker 1997). The drop assays on solid medium showed that all the transfected *E. coli* grew well on the selective medium with high K^+^ level, indicating a successful transfection of these vectors. Interestingly, when grown on the medium with low K^+^ level, only the *E. coli* transfected with *OsGLR2.1* and *OsGLR3.2* survived, indicating a complementation of OsGLR2.1 and OsGLR3.2 to the K^+^ uptake-deficiency of LB650 (Additional file 7B).

### Subcellular Localization of OsGLRs Expressed in Plant and Mammalian Cells

**Fig. 6:**
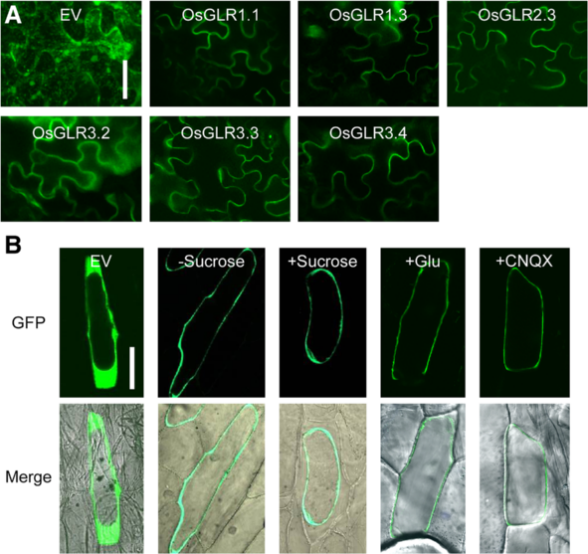
Subcellular localization of OsGLRs in plant cells. **a** GFP fluorescence images of tobacco leaf epidermal cells expressing *OsGLR-GFP*. Bar = 50 μm. **b** GFP fluorescence images of onion epidermal cells expressing *OsGLR1.1-GFP*. GFP fluorescence images before (−Sucrose) and after plasmolysis (+Sucrose) are shown. Treatments of Glu (20 mM for half an hour) and CNQX (0.1 mM for half an hour) did not alter the GFP fluorescence in onion epidermal cells. Bar = 100 μm. EV, empty vector control

In order to investigate the subcellular localization of OsGLR proteins, the CDS (without stop codon) of seven *OsGLR* genes were cloned into *pGWB405* (Nakagawa et al. 2007) and *pDEST47* (Invitrogen) respectively. In all the vectors, the Green Fluorescent Protein (GFP) was fused to the C terminus of OsGLRs, and these vectors were transformed into plant cells and mammalian cells. Tobacco leaf epidermal cells expressing fusion proteins showed similar fluorescence at the cell periphery, indicating plasma membrane localizations of OsGLRs (Fig. 6a). To confirm our results, we transiently expressed OsGLR1.1-GFP in onion epidermal cells. After plasmolysis, the GFP signal was observed to localize on the plasma membrane (Fig. 6b). We also checked the GFP fluorescence after treated the onion epidermal cells with Glu and CNQX. We found that neither Glu nor CNQX treatment altered the GFP fluorescence in onion epidermal cells (Fig. 6b).

The adherent mammalian HEK293 cells expressing OsGLR-GFP fusions showed similar localization to the previously published example of AtGLR3.4-GFP fusion protein, which was detected on the plasma membrane as well as internal membranes in HEK cells (Vincill et al. 2012) (Additional file 8). In order to confirm the subcellular localization of OsGLRs in HEK cells, we treated the HEK cells with trypsin to depart the adherent HEK cells to petri dish. After the treatment, the flat irregular shaped adherent HEK cell turned to globose suspended cell, and the GFP signal was detected at the edge of the cell (Additional file 8 inserted).

### OsGLR2.1 Expression is Involved in Glu-Induced [Ca^2+^]_i_ Increase in HEK Cells

**Fig. 7:**
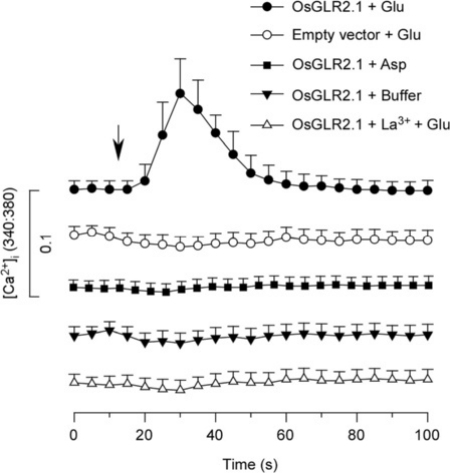
OsGLR2.1 expression is involved in Glu-induced [Ca^2+^]_i_ increase in HEK cells. [Ca^2+^]_i_ increase was analyzed by Fura-2 emission ratios (F340 nm:F380 nm). *Arrow* indicates the applications of Glu, Asp or buffer control. The baselines of different treatments were the same (F340 nm:F380 nm ≈ 0.136), and the lines were separated for clearness. Application of Glu to OsGLR2.1-expressing HEK cells was able to induce the [Ca^2+^]_i_ increase, while other treatments had no effect to change the [Ca^2+^]_i_ level in HEK cells. Fluorescence data were collected every 5 s. Data for independent cells in one experiment are shown (mean ± sd; *n* = 20)

HEK cells display little or no endogenous amino acid-gated channel activity (Zhang and Huganir 1999), and are a standard functional expression system for both mammalian iGluR and plant GLR channels (Chazot et al. 1999; Vincill et al. 2012). Much important knowledge about iGluRs and GLRs has been learned by studying them in HEK cells (Dravid et al. 2008; Keinanen et al. 1990; Monyer et al. 1992; Vincill et al. 2012; Vincill et al. 2013). To examine whether any of the newly isolated OsGLRs can mediate Glu-induced [Ca^2+^]_i_ increase in HEK cells, we examined the Glu-gated ion channel activities of OsGLRs in HEK cells. HEK cells were transfected with plasmids carrying OsGLR cDNAs, and analyzed their activity using Fura-2-based Ca^2+^ imaging. As a result, the application of Glu triggered a [Ca^2+^]_i_ increase in HEK cells expressing OsGLR2.1 (Additional file 9), and pretreatment of La^3+^, a Ca^2+^ channel blocker, inhibited the [Ca^2+^]_i_ increase. Furthermore, aspartate (Asp), which has similar chemical properties with Glu, was unable to induce a [Ca^2+^]_i_ increase in HEK cells expressing OsGLR2.1 (Fig. 7). This showed that at least OsGLR2.1 is able to mediate Glu-induced [Ca^2+^]_i_ increase in HEK cells. This also indicated that OsGLR2.1 may participate in the Glu-triggered Ca^2+^ influx in rice roots.

## Discussion

Glu is the primary natural ligand of iGluRs in the central nervous system in animals (Traynelis et al. 2010). In this research, the effectiveness of different amino acids was tested using the rice expressing aequorin. Of all the amino acids tested, Glu was clearly the most effective agonist to evoke the [Ca^2+^]_i_ increase in rice roots. This is different from the previous reports that a broad range of agonist of amino acids were able to induce Ca^2+^ responses in *Arabidopsis* (Qi et al. 2006; Vincill et al. 2012; Zhu et al. 2013). We tested the six amino acids which were reported to induce Ca^2+^ responses in aequorin-based luminescence system in *Arabidopsis* (Zhu et al. 2013). None of them, except Glu, induced induce Ca^2+^ in rice. It is worth noting that our aequorin-based luminescence imaging system was only able to reflect the [Ca^2+^]_i_ level in rice roots in contrast with the whole seedlings in *Arabidopsis* (Zhang et al. 2015). It is possible that other amino acids instead of Glu are able to induce [Ca^2+^]_i_ increase in rice shoots. Alternatively, rice may have evolved different approaches to cope with amino acid signals in different environmental conditions after the divergence of monocots and dicots. It was reported that acidic pH could also cause [Ca^2+^]_i_ increase (Zhu et al. 2013). In our experiment, we used glutamic acid monosodium salt solution for glutamate treatments, and the pH value was very close to 7 and would not cause a pH effect in our experiment. In addition, according to our time course experiment, the [Ca^2+^]_i_ response was very rapid (within 20 s). This is different from the pH effect, which lasts for several minutes (Zhu et al. 2013). According to our previous results, 100 mM of NaCl had no significant effect to [Ca^2+^]_i_ increase in rice roots (Zhang et al. 2015). While in our experiment, the concentration of Na^+^ was only 10 mM. Therefore, the [Ca^2+^]_i_ increase in rice roots was caused by glutamate, not by pH or salt stress effects.

We investigated the [Ca^2+^]_i_ changes in response to different concentrations of Glu and found that the response was very similar to previously reported [Ca^2+^]_i_ changes in response to Glu in *Arabidopsis* leaves, which fitted well by the Michaelis-Menten function, indicating similar mechanisms between these two species (Meyerhoff et al. 2005). It is reported that the concentrations of individual amino acids in the bulk soil solution are in the 0.01–10 μM range (Jones et al. 2005). What’s more, the concentrations of amino acids in different tissues of plants are below 10 mM (Pilot et al. 2004). Considering that the Km value of fitted curve was 9.60 mM, which fall into the range of *in planta* Glu concentration, we propose that the Ca^2+^ signaling system may participate in a sensory mechanism for *in planta* Glu concentration. In the pharmacological tests of Glu-triggered [Ca^2+^]_i_ increase, LaCl_3_ and GdCl_3_ inhibited about 50 % of [Ca^2+^]_i_ increase, and no significant differences were observed among different concentrations of blockers, indicating a saturation of Ca^2+^ channels blocked by LaCl_3_ and GdCl_3_. This is different from the inhibition effect of NaCl and H_2_O_2_ induced [Ca^2+^]_i_ increases in rice roots, which reached almost 100 and 90 % respectively (Zhang et al. 2015). Moreover, research in Glu-triggered [Ca^2+^]_i_ increase in *Arabidopsis* leaves showed about 90 % of inhibition by LaCl_3_ (Meyerhoff et al. 2005). Stephens et al. (2008) presented a model of GLR channels based on three types of qualitatively different, heteromeric GLR channel subtypes. We reasoned that different GLRs are able to assemble a large number of different channels, and the sensitivities of these channels to GdCl_3_/LaCl_3_ may different. The relative weak effects of Ca^2+^ channel blockers compared to that of *Arabidopsis* may also be due to our calculation method of aequorin luminescence signal. In order to obtain better repeatability, we only chose the region of primary root to calculate the level of luminescence signal. After the pretreatment with GdCl_3_ and LaCl_3_, most of the luminescence signal came from the region of primary root, thus our method may result to the underestimate of the effects of these blockers. We also observed that CNQX and DNQX inhibited the Glu-triggered [Ca^2+^]_i_ increase significantly. CNQX and DNQX were initially used to inhibit the activities of iGluR channels in animals (Armstrong and Gouaux 2000; Honore et al. 1988). Subsequent experiments showed that they were also efficient inhibitors of GLRs in plants (Dubos et al. 2003; Manzoor et al. 2013; Michard et al. 2011; Vatsa et al. 2011). It was also shown that the efficiency of these inhibitors was about 50 %, which is similar to our results (Vatsa et al. 2011).

Ca^2+^ channels in plants belong to large gene families, and these channels are regulated by different effectors (Jammes et al. 2011). For instance, mechanosensitive Ca^2+^-permeable channels (MSCC) are controlled by mechanical stimulation (Monshausen and Gilroy 2009), cyclic nucleotide gated channels (CNGC) are controlled by cyclic nucleotides (Kaplan et al. 2007), two-pore channels (TPC) are controlled by membrane potential (Pottosin and Schonknecht 2007) and GLR is controlled by amino acids (Price et al. 2012). In our research, we found that Glu specifically triggered [Ca^2+^]_i_ increase in rice roots. Together with the desensitization assay and pharmacological tests, we propose that the Glu-triggered [Ca^2+^]_i_ increase in rice roots is mediated by OsGLR family. Compared with 20 *AtGLR* genes in *Arabidopsis* (Chiu et al. 2002; Davenport 2002), the number of *OsGLR* genes in rice is still uncertain. It was stated that there are 13 *OsGLR* genes in rice (Ward et al. 2009). However, it was also demonstrated that rice has at least 21 *OsGLR* genes (ARAMEMNON plant membrane protein database: release 7) (Forde 2014). In this research, we chose a total of 13 *OsGLR* genes that have the closest relationship to the *Arabidopsis* 20 *AtGLR* genes for further analysis. According to the phylogenetic tree, we found that *GLRs* from *Arabidopsis* and rice separated to each other in clade I and clade II, which indicated independent evolution of GLRs in these clades after the divergence of monocots and dicots.

It is reported that all 20 *AtGLR* genes are expressed in *Arabidopsis*, and there are no distinct clade-specific organ expression patterns among the three *AtGLR* clades (Chiu et al. 2002). On the basis of our qRT-PCR experiment, we found expression diversities among members of the *OsGLR* family. It is worth noting that members in clade III had relatively higher expression levels compared with the other two clades, except for *OsGLR3.1*, which had very low expression levels in all the tissues examined. It is also reported that all 20 *AtGLR* genes are expressed in roots and five of the nine clade II genes are root-specific in *Arabidopsis* (Chiu et al. 2002). Similarly, most of the *OsGLR* genes had higher expression levels in roots. Furthermore, two of the four clade II genes (*OsGLR2.1* and *OsGLR2.3*) are almost root-specific in rice. It is worth noting that all four *OsGLRs* in clade II are tandemly clustered in Chromosome 9, and the expression patterns of *OsGLR2.1* and *OsGLR2.3* are very similar to each other, with the highest level in the roots. This indicates that OsGLR2.1 and OsGLR2.3 may have similar functions in the roots.

For further analysis, we cloned seven of the *OsGLRs* from those which had the higher expression levels. So far, the strongest phenotype reported for a single *glr* mutant is in rice which is defective in the *OsGLR3.1* gene (Li et al. 2006). Interestingly, the expression level of *OsGLR3.1* is the lowest in clade III. Thus, for full understanding of the OsGLR family functions in rice, it is necessary to clone all the *OsGLR* genes for functional analysis in future, although some of them have very low expression levels.

In the bacteria growth assay, *E. coli* transfected with Entry clones inserted with *OsGLR2.1*, *OsGLR2.3* and *OsGLR3.2* had a slower growth rate than the others. The Entry clones have a background of *pDONR201*, which have no known promoters beside the insert region. It is possible that other elements (T1 and T2 sequences for instance) are responsible for the leaky expression of *OsGLR2.1*, *OsGLR2.3* and *OsGLR3.2*, which are enough to affect bacteria growth. In the plant cell subcellular localization analysis of OsGLR2.1, we failed to shuttle *OsGLR2.1* into the destination vector *pGWB405* by LR reaction. It was reported that 35S promoter can also be activated in *E. coli* (Assaad and Signer 1990). *OsGLR2.1* driven by 35S may have a more serious leaky effect, which results in the excessive ion channel activities and subsequently leads to the death of the transfected *E. coli*. We did not add extra glutamate into the medium in both bacteria growth assays. It is possible that tryptone and yeast extract in the medium contain a small amount of glutamate, which is sufficient to operate the channels formed by OsGLRs. Considering the non-selective nature of GLR channels in *Arabidopsis* (Jammes et al. 2011), our bacterial results showed that OsGLRs may also form non-selective channels at least in *E. coli*.

We expressed OsGLR-GFP fusion proteins in tobacco leaf epidermal cells and onion epidermal cells to examine the subcellular localizations of OsGLRs. As a result, all the members examined had similar subcellular localization patterns. We observed that the GFP signal was detected at the cell periphery when expressed in both the tobacco leaf epidermal cells and onion epidermal cells, which indicated that these OsGLR proteins are very likely plasma membrane localized proteins. Until now, to our knowledge, many of the AtGLRs examined are plasma membrane localized proteins (Meyerhoff et al. 2005; Tapken et al. 2013; Vincill et al. 2012; Vincill et al. 2013). However, interestingly, some AtGLRs were also reported to localize to other organelles. It was reported that AtGLR3.4 has a dual localizations to plastids and plasma membrane (Teardo et al. 2011). Splicing variant of AtGLR3.5 targets the inner mitochondrial membrane, while the other variant localizes to the chloroplasts (Teardo et al. 2015). From this point of view, although plasmolysis analysis in onion epidermal cells provided additional evidence of plasma membrane localization of OsGLRs, further experiments are still needed (Co-localization analysis by a plasma membrane marker for instance) to provide unequivocal evidence of subcellular localizations of OsGLR proteins. We also expressed OsGLR-GFP fusion proteins in mammalian HEK cells, and the GFP fluorescence was detected on the plasma membrane as well as internal membranes in HEK cells. The altered subcellular localization may probably due to the different systems between plant and animal cells. The HEK cell results also showed that the OsGLRs can be expressed in animal cells and a Ca^2+^ imaging approach in HEK cells was worth pursuing.

We screened the Ca^2+^ channel activities of these newly isolated OsGLRs in HEK cell system and found that OsGLR2.1 was able to mediate Glu-triggered [Ca^2+^]_i_ increase in HEK cells. Furthermore, pretreatment of a Ca^2+^ channel blocker inhibited the increase. We found that the change in Fura-2 ratio in OsGLR2.1-expresing cell is relatively small. We reasoned that this may due to the heterologous expression of OsGLR2.1 in HEK cells. Alternatively, OsGLR2.1 may need to interact with other partners to from efficient ion channels. Our results indicated that OsGLR2.1 is able to form a Ca^2+^ permeable channel in HEK cells. Considering the root expression of *OsGLR2.1*, It could be responsible for Glu-triggered [Ca^2+^]_i_ increase in rice roots. However, it is not necessarily that OsGLR2.1 is sufficient to assume the Glu-triggered [Ca^2+^]_i_ increase in rice roots. It is possible that rice roots may employ more complex GLR channels rather than those formed by OsGLR2.1. Animal iGluRs often combine as heterotetramers to form amino acid gated ion channels (Traynelis et al. 2010), and it is generally assumed that plant GLRs will assemble in a similar way (Forde 2014). Using desensitization analysis and knock out mutant, the model of different AtGLR subtypes was proposed (Stephens et al. 2008). Furthermore, the interactions between different subunits were also reported (Price et al. 2013). HEK cells transfected with *AtGLR3.4* alone showed amino acid triggered ion channel activities (Vincill et al. 2012). Further research showed that AtGLR3.4 physically interacts with AtGLR3.2 to form heteromeric ion channels to affect lateral root development in the phloem (Vincill et al. 2013). From this point of view, it is possible that OsGLR2.1 may interact with other OsGLRs to from heteromeric ion channels in rice roots.

## Conclusions

We described a Glu-specific triggered [Ca^2+^]_i_ increase in rice roots. A desensitization assay and pharmacological tests showed that this response is mediated by OsGLR channels. We identified 13 *OsGLRs* and cloned seven of them. Further research showed that OsGLR2.1 and OsGLR3.2 have the potential to mediate ion uptake in bacteria, and HEK cells expressing OsGLR2.1 are able to permeate Ca^2+^ that are controlled by Glu. Our results indicated that OsGLR2.1 may participate in the Glu-triggered Ca^2+^ influx in rice roots. For future directions, genetic analysis (loss of function of *osglr2.1* mutant) and protein-protein interaction assays (the OsGLRs interact with OsGLR2.1) are needed to demonstrate the mechanism of Glu-triggered Ca^2+^ influx in rice roots.

## Methods

### Aequorin Luminescence Imaging

Seeds of aequorin-expressing rice were sown and grown on plates as described previously (Zhang et al. 2015). Reconstitution of aequorin was performed in vivo by spraying transgenic seedlings with 10 μM coelenterazine and followed by incubation at 21 °C in the dark for 12–16 h. Treatments and aequorin luminescence imaging were performed at room temperature using a ChemiPro HT system as described previously (Jiang et al. 2013). For screening of the specificity of amino acids in induction of Ca^2+^ flux in rice roots, 10 mM amino acid solutions (Glutamate, Sigma G1626; Glutamine, Sigma G3126; Glycine, Sigma G7126; Cysteine, Sigma C1276; Alanine, Sigma A7627; Serine, Sigma S4500; Asparagine, Sigma A8381; Aspartic acid, Sigma A9256; Methionine, Sigma M9625) were used and luminescence images were acquired for 3 min. For the analysis of time courses of increase in [Ca^2+^]_i_, each exposure time was 20 s and the images were taken continuously for 3 min. Water was used instead of amino acid solution in the control experiment. The fitted curve was calculated by SigmaPlot 12.5. For desensitization analysis, plants were first treated with 10 mM Glu for aequorin luminescence imaging. After the imaging, plants were washed with water several times to remove the residual Glu and transferred to a new petri dish. The plants were treated with or without 0.1 mM of CHX and placed in the dark for 1 h. The second application of 10 mM Glu was carried out after 1 h. For pharmacological tests, rice roots were treated with different concentrations of LaCl_3_, GdCl_3_, CNQX and DNQX, respectively for 30 min before 10 mM Glu treatment was applied. CNQX and DNQX were first dissolved in DMSO (due to the limited solubility, the concentration of stock solutions were 20 mM), the stock solutions were then diluted with water to final concentrations. For calculations of average luminescence intensity, luminescence signal in the region of primary roots were manually and equivalently selected, and the average luminescence intensity was analyzed. To avoid the interference of chloroplast auto-fluorescence signal in the aequorin luminescence imaging, all the treatments were performed in complete darkness. WinView/32 and Meta Morph 7.7 were used to analyze recorded luminescence images.

### Identification of *OsGLR* Gene Family in Rice

To identify potential members of the *OsGLR* gene family in rice, we performed multiple database searches. Twenty *Arabidopsis AtGLR* sequences (Chiu et al. 2002) were used as queries in BLAST searches against the rice genome sequence (http://www.phytozome.net/). The Hidden Markov Model (HMM) profiles of the GLR family (Pfam 01094: Receptor family ligand binding region; Pfam 00060: Ligand-gated ion channel; Pfam 00497: Bacterial extracellular solute-binding proteins, family 3; Pfam 13458: Periplasmic binding protein) were used to confirm the OsGLR gene family in rice.

### Phylogenetic Tree Building, Transmembrane Helices and Subcellular Localization Predictions

Multiple sequence alignments were performed on the AtGLR and OsGLR proteins using ClustalW with default parameters, and the alignments were then adjusted manually. The phylogenetic tree was constructed using MEGA6.06 employing the Maximum-Likelihood (ML) method. To construct better phylogenetic tree, we added 61 GLRs form poplar (Ward et al. 2009) along with GLRs from rice and *Arabidopsis*. The CDS of *OsGLRs* were mostly obtained from a public database (http://rice.plantbiology.msu.edu/), some sequences were modified based on our sequencing data. The transmembrane domains were estimated using TMHMM2 (www.cbs.dtu.dk/services/TMHMM/). The subcellular localization predictions were estimated using PSORT (http://wolfpsort.org/).

### RNA Isolation and Quantitative RT-PCR

Total RNA from different tissues including shoots, stem-base and roots of 5 day old seedlings, and the stem and young panicle of adult plants were extracted using a Plant RNeasy Mini kit (Qiagen) according to the manufacturer’s instructions. First-strand cDNA was synthesized with SuperScript first-strand synthesis kit (Invitrogen) and the transcript levels of each gene were measured by qRT-PCR using Mx3000p QPCR system (Agient) with iQ SYBR Green Supermix (Bio-Rad). The program for the qRT-PCR was as follows: 5 min at 95 °C, and 45 cycles of 15 s at 95 °C, 20 s at 60 °C, 20 s at 72 °C. The heat map representation was performed according to the previous description (Shen et al. 2014). Briefly, the 1/1000 of *OsACTIN* expression level was considered as an internal standard to calculate the relative fold differences based on the comparative cycle threshold (2^-∆∆Ct^) values. The logarithm of expression level change fold data were used by Treeview to visualize as a heat map. Red color represents higher expression level, black represents similar expression level and green color represents lower expression level. All the expression analysis was carried out for at least three biological repeats and the values shown in figures represents the average values of these repeats. For the expression analysis of different *OsGLRs* after treatment with Glu, rice roots were treated with 20 mM Glu for different time phrases. For the inhibitor pretreatment, rice roots were treated with 0.1 mM CNQX for half an hour. The expression analysis was carried out for three biological repeats. The primers used in this experiment were listed in Additional file 10.

### Cloning and Recombinant Vector Construction

Full-length CDS (with or without stop codon) of *OsGLRs* were isolated from total RNA by RT-PCR and amplified using KOD-FX DNA polymerase (TOYOBO); the primers were listed in Additional file 10. The PCR products were cloned into the *pDONR201* entry vector using the BP Clonase™ Enzyme Mix (Invitrogen) according to the manufacturer’s instructions. For the complementation of LB650 and the imaging of [Ca^2+^]_i_ in HEK 293 cells, the *pDONR201* entry vector containing CDS (with stop codon) of *OsGLRs* were shuttled into the Gateway destination vectors *pDEST14* and *pcDNA3.2* respectively using the LR Clonase™ Enzyme Mix (Invitrogen) according to the manufacturer’s instructions. To avoid the toxic effect of *ccdB* gene to the *E.coli*, the CDS of *GFP* was used to replace the poison gene *ccdB*, and the vectors containing *GFP* were considered as empty vectors in the measurement of bacteria growth rate and complementation of LB650. For the subcellular localization analysis of OsGLRs in the plant and mammalian cells, the *pDONR201* entry vector containing CDS (without stop codon) of *OsGLRs* were shuttled into the Gateway destination vectors *pGWB405* (Nakagawa et al. 2007) and *pDEST47* (Invitrogen) by LR reactions respectively, which fuse the GFP tag to the C terminus of the translated gene product. The *pcDNA3.2* containing *GFP* was considered as an empty vector in the subcellular localization analysis in the mammalian cells. For the empty vector in the subcellular localization analysis in the plant cells, CDS of *GFP* was shuttled into the Gateway destination vector *pGWB402* (Nakagawa et al. 2007) by LR reaction, which had a cauliflower mosaic virus (CaMV) 35S promoter to drive the *GFP* gene.

### Bacterial Grown Assay

For the measurement of bacteria growth rate, transformants were first grown at 37 °C in a shaking incubator with 3 ml liquid LB medium containing 10 g/L tryptone, 10 g/L NaCl and 5 g/L yeast extract. After approximately 12–16 h of growth, we adjusted all the cultures to the same density. Cell culture (1 ml) with a density of 0.6 OD_600_ was added to 100 ml LB medium and continued to grow at 37 °C and 220 rpm. The cell density was measured 12 times at 1 h intervals after the transfer. *GFP* gene was cloned into *pDONR201* to act as the empty vector comparison.

For the complementation of a K^+^-uptake mutant of *E. coli* by OsGLRs, the K^+^ uptake-deficient *E. coli* strain LB650 with mutations in the *TRK* H and *TRK* G genes was used for complementation studies (Stumpe and Bakker 1997). The *pDEST14* containing with *OsGLRs* (or *GFP* as empty vector) were transformed into the *E. coli* LB650 strain. The complementation assay was carried out as previously reported (Ali et al. 2006).

### HEK Cell Culture and Transfection

HEK293T cells were grown and maintained in DMEM medium (GIBCO) supplemented with 10 % (*v/v*) fetal bovine serum (GIBCO) and 0.1 % (*w/v*) Penicillin-Streptomycin antibiotics (GIBCO), and placed in a 37 °C incubator with 95 % (*v/v*) air and 5 % (*v/v*) CO_2_. For transfection, cells were first seeded onto poly-lysine-coated eight-well chambered coverglass (Nunc) and cultured with DMEM medium supplemented with 5 % (*v/v*) fetal bovine serum. After growth for 24 h, cells were transfected with plasmid DNA using Lipofectamine 2000 reagent (Invitrogen) as described previously (Yuan et al. 2014). The transfected HEK cells were grown for additional 24 h before subsequent experiments.

### Subcellular Localization Analysis of OsGLR Proteins

For subcellular localization analysis in tobacco leaf epidermal cells, *Agrobacterium tumefaciens*–mediated transient transformation of *N. benthamiana* was performed as described (Li et al. 2008; Voinnet et al. 2003). Briefly, *A. tumefaciens* strain EHA105 carrying binary constructs was grown to OD_600_ values of 0.2 to 0.4 at 28 °C in LB medium. Agrobacterium cells were collected by centrifugation at 4000 rpm for 10 min at 25 °C, and resuspended in 10 mM MES (pH 5.7), 10 mM MgCl_2_ and 150 mg/ml acetosyringone. Leaves of 4–5-week-old plants were infiltrated with bacterial cultures, and the fluorescence of GFP was observed under a Nikon A1 confocal microscope. For subcellular localization analysis in onion epidermal cells, plasmid-coated gold microparticles (Φ = 1.0 μm) were accelerated by helium pressure (1100 psi) propeling the macrocarrier to onion by PDS-1000/He system (Bio-Rad, USA). The onion was then incubated at 26 °C in the dark. After 16 h cultivation, the onion epidermis was imaged by confocal laser scanning microscope (Nikon A1). The 0.3 g/ml of sucrose was used in plasmolysis, the 20 mM of Glu was used in Glu treatment (treated for half an hour) and the 0.1 mM of CNQX was used in blocker treatment (treated for half an hour).

For subcellular localization analysis of OsGLR proteins in HEK cells, cells transfected with plasmid DNA were grown in poly-lysine-coated eight-well chambered coverglass for 24 h and the fluorescence of GFP was observed by Zeiss Axio Observer A1 fluorescence microscope directly. To confirm the subcellular localization of OsGLRs in HEK cells, the HEK cells were treated with trypsin (GIBCO) and incubated for 5 min. After the departure of HEK cells, the trypsin was replaced with PBS (GIBCO) and the globose suspended HEK cells were observed for subcellular localizations.

### Imaging of [Ca^2+^]_i_ in HEK Cells

HEK cells transfected with plasmid DNA were loaded with Ca^2+^ sensitive dye Fura-2 AM (Sigma), and a Fura-2 based Ca^2+^ imaging assay was performed using the Axiovert 200 fluorescence microscope. Emission ratiometric images (F340 nm:F380 nm) were collected using MetaFluor Fluorescence Ratio Imaging Software (Molecular Devices). For the Glu treatments, Fura-2 loaded HEK293 cells were incubated in a standard buffer (pH 7.4) containing 130 mM NaCl, 3 mM KCl, 0.6 mM MgCl_2_, 10 mM glucose, 10 mM HEPES and 1 mM Ca^2+^ for 30 min. One hundred microliter Glu solution (30 mM) was dropped carefully into the 200 μl standard buffer (the final concentration of Glu was 10 mM), and the Fura-2 ratiometric images were collected. For Ca^2+^ channel blocker treatment, HEK cells were pretreated with LaCl_3_ (1 mM) and incubated for 30 min before the Glu treatments. For control treatments, standard buffer solution and Asp solution were used instead of Glu. For data analysis, about 20 cells in one experiment were selected manually based on the increases in [Ca^2+^]_i_. All the experiments were repeated for at least three times.

## Additional Files

Additional file 1: Desensitization of Glu activated Ca^2+^ channels in rice roots. (A) Pseudocolor images of aequorin luminescence in response to two sequential Glu treatments. From left to right, the first application of Glu (left), the second application of Glu after1 hour’s recovery (middle) and second application of Glu pretreated with CHX (right). The relationship between luminescence intensity and the pseudocolor images are scaled by a pseudocolor bar and the numbers next to the pseudocolor bar are maximum and minimum values of luminescence intensity. (B) The bar chart of relative luminescence signal intensity of every treatment. The results were obtained from at least three independent experiments (mean ± sd; *n* = 10). (TIF 1537 kb) Additional file 2: The effect of DMSO in the Glu-induced [Ca^2+^]_i_ increase. (A) Pseudocolor images of aequorin luminescence in roots treated by different percentages of DMSO. The relationship between luminescence intensity and the pseudocolor images are scaled by a pseudocolor bar and the numbers next to the pseudocolor bar are maximum and minimum values of luminescence intensity. (B) Relative luminescence signal intensity of every treatment. The results were obtained from at least three independent experiments (mean ± sd; *n* = 10; NS, not significant *P* > 0.05; Student’s *t*-test). (TIF 2858 kb) Additional file 3: The information of *OsGLR* genes in rice. (XLSX 12 kb) Additional file 4: Transmembrane helices structure predictions of OsGLR proteins. The transmembrane domains were estimated using TMHMM2: www.cbs.dtu.dk/services/TMHMM/. The red peaks showed the predicted transmembrane regions of proteins; the blue peaks showed the inside transmembrane domains; the pink peaks showed the outside transmembrane domain. (TIF 625 kb) Additional file 5: The sequence alignment of 20 AtGLRs and 13 OsGLRs (PDF 1229 kb) Additional file 6: qRT-PCR analysis of *OsGLRs* after treatment of Glu and CNQX. For Glu treatment, rice roots were treated with 20 mM Glu for 5 min or 30 min. For blocker treatment, rice roots were treated with 0.1 mM CNQX for 30 min. In addition, rice roots were also pretreated with 0.1 mM CNQX for 30 min, then treated with 20 mM Glu for 30 min (CNQX + Glu). Data for independent experiments are shown (mean ± sd; *n* = 3). (TIF 189 kb) Additional file 7: OsGLRs mediate ion uptake in bacteria. (A) Bacterial growth rate of *E. coli*, transfected with different *OsGLRs*. Expression of OsGLR2.1, OsGLR2.3 and OsGLR3.2 reduced the growth rate of *E. coli* compared with the empty vector control. (B) K^+^ uptake-deficient *E. coli* mutant (strain LB650) transformed with different *OsGLRs*, was grown on solid selective medium with either high K^+^ (100 mM) or low K^+^ (2 mM). Expression of OsGLR2.1 and OsGLR3.2 enhance the growth of LB650 on low K^+^ medium compared with the empty vector control. (TIF 1891 kb) Additional file 8: Subcellular localization of OsGLRs in HEK293 cells. GFP fluorescence images of HEK293 cells expressing *OsGLR-GFP*. HEK cells treated with trypsin were inserted with red frames. EV, empty vector control. Bar = 10 μm. (TIF 1119 kb) Additional file 9: The application of Glu triggered a [Ca^2+^]_i_ increase in HEK cells expressing OsGLR2.1 (movie in the ppt file). (PPTX 19380 kb) Additional file 10: The sequences of primers used in this research. (XLSX 13 kb)

## Abbreviations

Asn: asparagine
Asp: aspartate
[Ca^2+^]_i_: concentration of cytosolic free Ca^2+^
CDS: coding sequence
CHX: cycloheximide
CNQX: 6-Cyano-7-Nitroquinoxaline-2,3-dione
Cys: cysteine
DMSO: dimethyl sulfoxide
DNQX: 6,7-Dinitroquinoxaline-2,3-dione
GFP: green fluorescent protein
GLR: GLUTAMATE RECEPTOR-LIKE
Glu: glutamate
Gly: glycine
HEK: human embryonic kidney
HMM: hidden Markov model
iGluR: ionotropic glutamate receptor
LB: Luria Bertani
Met: Methionine
Mw: molecular weight
NJ: neighbor-joining
pI: isoelectric point
qRT-PCR: quantitative reverse transcription polymerase chain reaction
Ser: serine
TMH: transmembrane helices

## References

[CR1] Ali R, Zielinski RE, Berkowitz GA (2006). Expression of plant cyclic nucleotide-gated cation channels in yeast. J Exp Bot.

[CR2] Armstrong N, Gouaux E (2000). Mechanisms for activation and antagonism of an AMPA-sensitive glutamate receptor: crystal structures of the GluR2 ligand binding core. Neuron.

[CR3] Assaad FF, Signer ER (1990). Cauliflower mosaic virus P35S promoter activity in Escherichia coli. Mol Gen Genet.

[CR4] Chazot PL, Cik M, Stephenson FA (1999). Transient expression of functional NMDA receptors in mammalian cells. Methods Mol Biol.

[CR5] Chiu J, DeSalle R, Lam HM, Meisel L, Coruzzi G (1999). Molecular evolution of glutamate receptors: a primitive signaling mechanism that existed before plants and animals diverged. Mol Biol Evol.

[CR6] Chiu JC, Brenner ED, DeSalle R, Nitabach MN, Holmes TC, Coruzzi GM (2002). Phylogenetic and expression analysis of the glutamate-receptor-like gene family in Arabidopsis thaliana. Mol Biol Evol.

[CR7] Davenport R (2002). Glutamate receptors in plants. Ann Bot.

[CR8] Dennison KL, Spalding EP (2000). Glutamate-gated calcium fluxes in Arabidopsis. Plant Physiol.

[CR9] Dingledine R, Borges K, Bowie D, Traynelis SF (1999). The glutamate receptor ion channels. Pharmacol Rev.

[CR10] Dravid SM, Prakash A, Traynelis SF (2008). Activation of recombinant NR1/NR2C NMDA receptors. J Physiol.

[CR11] Dubos C, Huggins D, Grant GH, Knight MR, Campbell MM (2003). A role for glycine in the gating of plant NMDA-like receptors. Plant J.

[CR12] Forde BG (2014). Glutamate signalling in roots. J Exp Bot.

[CR13] Honore T, Davies SN, Drejer J, Fletcher EJ, Jacobsen P, Lodge D, Nielsen FE (1988). Quinoxalinediones: potent competitive non-NMDA glutamate receptor antagonists. Science.

[CR14] Jammes F, Hu HC, Villiers F, Bouten R, Kwak JM (2011). Calcium-permeable channels in plant cells. FEBS J.

[CR15] Jiang Z, Zhu S, Ye R, Xue Y, Chen A, An L, Pei ZM (2013). Relationship between NaCl- and H_2_O_2_-Induced Cytosolic Ca^2+^ Increases in Response to Stress in *Arabidopsis*. PLoS ONE.

[CR16] Jones DL, Shannon D, Junvee-Fortune T, Farrar JF (2005). Plant capture of free amino acids is maximized under high soil amino acid concentrations. Soil Biol Biochem.

[CR17] Jones MV, Westbrook GL (1996). The impact of receptor desensitization on fast synaptic transmission. Trends Neurosci.

[CR18] Kang J, Mehta S, Turano FJ (2004). The putative glutamate receptor 1.1 (AtGLR1.1) in Arabidopsis thaliana regulates abscisic acid biosynthesis and signaling to control development and water loss. Plant Cell Physiol.

[CR19] Kang J, Turano FJ (2003). The putative glutamate receptor 1.1 (AtGLR1.1) functions as a regulator of carbon and nitrogen metabolism in Arabidopsis thaliana. Proc Natl Acad Sci U S A.

[CR20] Kaplan B, Sherman T, Fromm H (2007). Cyclic nucleotide-gated channels in plants. FEBS Lett.

[CR21] Keinanen K, Wisden W, Sommer B, Werner P, Herb A, Verdoorn TA, Sakmann B, Seeburg PH (1990). A family of AMPA-selective glutamate receptors. Science.

[CR22] Kong D, Ju C, Parihar A, Kim S, Cho D, Kwak JM (2015). Arabidopsis glutamate receptor homolog3.5 modulates cytosolic Ca^2+^ level to counteract effect of abscisic acid in seed germination. Plant Physiol.

[CR23] Lacombe B, Becker D, Hedrich R, DeSalle R, Hollmann M, Kwak JM, Schroeder JI, Le Novere N, Nam HG, Spalding EP, Tester M, Turano FJ, Chiu J, Coruzzi G (2001). The identity of plant glutamate receptors. Science.

[CR24] Lam HM, Chiu J, Hsieh MH, Meisel L, Oliveira IC, Shin M, Coruzzi G (1998). Glutamate-receptor genes in plants. Nature.

[CR25] Li J, Zhu S, Song X, Shen Y, Chen H, Yu J, Yi K, Liu Y, Karplus VJ, Wu P, Deng XW (2006). A rice glutamate receptor-like gene is critical for the division and survival of individual cells in the root apical meristem. Plant Cell.

[CR26] Li X, Chanroj S, Wu Z, Romanowsky SM, Harper JF, Sze H (2008). A distinct endosomal Ca^2+^/Mn^2+^ pump affects root growth through the secretory process. Plant Physiol.

[CR27] Manzoor H, Kelloniemi J, Chiltz A, Wendehenne D, Pugin A, Poinssot B, Garcia-Brugger A (2013). Involvement of the glutamate receptor AtGLR3.3 in plant defense signaling and resistance to Hyaloperonospora arabidopsidis. Plant J.

[CR28] Meyerhoff O, Muller K, Roelfsema MR, Latz A, Lacombe B, Hedrich R, Dietrich P, Becker D (2005). AtGLR3.4, a glutamate receptor channel-like gene is sensitive to touch and cold. Planta.

[CR29] Michard E, Lima PT, Borges F, Silva AC, Portes MT, Carvalho JE, Gilliham M, Liu LH, Obermeyer G, Feijo JA (2011). Glutamate receptor-like genes form Ca^2+^ channels in pollen tubes and are regulated by pistil D-serine. Science.

[CR30] Miller ND, Durham Brooks TL, Assadi AH, Spalding EP (2010). Detection of a gravitropism phenotype in glutamate receptor-like 3.3 mutants of Arabidopsis thaliana using machine vision and computation. Genetics.

[CR31] Monshausen GB, Gilroy S (2009). Feeling green: mechanosensing in plants. Trends Cell Biol.

[CR32] Monyer H, Sprengel R, Schoepfer R, Herb A, Higuchi M, Lomeli H, Burnashev N, Sakmann B, Seeburg PH (1992). Heteromeric NMDA receptors: molecular and functional distinction of subtypes. Science.

[CR33] Mousavi SAR, Chauvin A, Pascaud F, Kellenberger S, Farmer EE (2013). GLUTAMATE RECEPTOR-LIKE genes mediate leaf-to-leaf wound signalling. Nature.

[CR34] Nakagawa T, Suzuki T, Murata S, Nakamura S, Hino T, Maeo K, Tabata R, Kawai T, Tanaka K, Niwa Y, Watanabe Y, Nakamura K, Kimura T, Ishiguro S (2007). Improved Gateway binary vectors: high-performance vectors for creation of fusion constructs in transgenic analysis of plants. Biosci Biotechnol Biochem.

[CR35] Pilot G, Stransky H, Bushey DF, Pratelli R, Ludewig U, Wingate VP, Frommer WB (2004). Overexpression of GLUTAMINE DUMPER1 leads to hypersecretion of glutamine from Hydathodes of Arabidopsis leaves. Plant Cell.

[CR36] Pottosin II, Schonknecht G (2007). Vacuolar calcium channels. J Exp Bot.

[CR37] Price MB, Jelesko J, Okumoto S (2012). Glutamate receptor homologs in plants: functions and evolutionary origins. Front Plant Sci.

[CR38] Price MB, Kong D, Okumoto S (2013). Inter-subunit interactions between glutamate-like receptors in Arabidopsis. Plant Signal Behav.

[CR39] Qi Z, Stephens NR, Spalding EP (2006). Calcium entry mediated by GLR3.3, an Arabidopsis glutamate receptor with a broad agonist profile. Plant Physiol.

[CR40] Shen C, Yue R, Yang Y, Zhang L, Sun T, Xu L, Tie S, Wang H (2014). Genome-wide identification and expression profiling analysis of the Aux/IAA gene family in Medicago truncatula during the early phase of Sinorhizobium meliloti infection. PLoS One.

[CR41] Stephens NR, Qi Z, Spalding EP (2008). Glutamate receptor subtypes evidenced by differences in desensitization and dependence on the GLR3.3 and GLR3.4 genes. Plant Physiol.

[CR42] Stumpe S, Bakker EP (1997). Requirement of a large K^+^-uptake capacity and of extracytoplasmic protease activity for protamine resistance of Escherichia coli. Arch Microbiol.

[CR43] Tapken D, Anschutz U, Liu LH, Huelsken T, Seebohm G, Becker D, Hollmann M (2013). A plant homolog of animal glutamate receptors is an ion channel gated by multiple hydrophobic amino acids. Sci Signal.

[CR44] Teardo E, Carraretto L, De Bortoli S, Costa A, Behera S, Wagner R, Lo Schiavo F, Formentin E, Szabo I (2015). Alternative Splicing-Mediated Targeting of the Arabidopsis GLUTAMATE RECEPTOR3.5 to Mitochondria Affects Organelle Morphology. Plant Physiol.

[CR45] Teardo E, Formentin E, Segalla A, Giacometti GM, Marin O, Zanetti M, Lo Schiavo F, Zoratti M, Szabo I (2011). Dual localization of plant glutamate receptor AtGLR3.4 to plastids and plasmamembrane. Biochim Biophys Acta.

[CR46] Tracy FE, Gilliham M, Dodd AN, Webb AA, Tester M (2008). NaCl-induced changes in cytosolic free Ca^2+^ in Arabidopsis thaliana are heterogeneous and modified by external ionic composition. Plant Cell Environ.

[CR47] Traynelis SF, Wollmuth LP, McBain CJ, Menniti FS, Vance KM, Ogden KK, Hansen KB, Yuan H, Myers SJ, Dingledine R (2010). Glutamate receptor ion channels: structure, regulation, and function. Pharmacol Rev.

[CR48] Vatsa P, Chiltz A, Bourque S, Wendehenne D, Garcia-Brugger A, Pugin A (2011). Involvement of putative glutamate receptors in plant defence signaling and NO production. Biochimie.

[CR49] Vincill ED, Bieck AM, Spalding EP (2012). Ca^2+^ conduction by an amino acid-gated ion channel related to glutamate receptors. Plant Physiol.

[CR50] Vincill ED, Clarin AE, Molenda JN, Spalding EP (2013). Interacting glutamate receptor-like proteins in Phloem regulate lateral root initiation in Arabidopsis. Plant Cell.

[CR51] Voinnet O, Rivas S, Mestre P, Baulcombe D (2003). An enhanced transient expression system in plants based on suppression of gene silencing by the p19 protein of tomato bushy stunt virus. Plant J.

[CR52] Ward JM, Maser P, Schroeder JI (2009). Plant ion channels: gene families, physiology, and functional genomics analyses. Annu Rev Physiol.

[CR53] Watkins JC, Jane DE (2006). The glutamate story. Br J Pharmacol.

[CR54] Yuan F, Yang H, Xue Y, Kong D, Ye R, Li C, Zhang J, Theprungsirikul L, Shrift T, Krichilsky B, Johnson DM, Swift GB, He Y, Siedow JN, Pei ZM (2014). OSCA1 mediates osmotic-stress-evoked Ca^2+^ increases vital for osmosensing in Arabidopsis. Nature.

[CR55] Zhang S, Huganir RL (1999). Calmodulin modification of NMDA receptors. Methods Mol Biol.

[CR56] Zhang Y, Wang Y, Taylor JL, Jiang Z, Zhang S, Mei F, Wu Y, Wu P, Ni J (2015). Aequorin-based luminescence imaging reveals differential calcium signalling responses to salt and reactive oxygen species in rice roots. J Exp Bot.

[CR57] Zhu X, Feng Y, Liang G, Liu N, Zhu JK (2013). Aequorin-based luminescence imaging reveals stimulus- and tissue-specific Ca^2+^ dynamics in Arabidopsis plants. Mol Plant.

