# Supplementary figures and images for: Heterologous Expression and Functional Analysis of Rice GLUTAMATE RECEPTOR-LIKE Family Indicates its Role in Glutamate Triggered Calcium Flux in Rice Roots
Source: Rice (N Y). 2016 Mar 8;9:9. doi: 10.1186/s12284-016-0081-x (PMC4783324; doi:10.1186/s12284-016-0081-x)

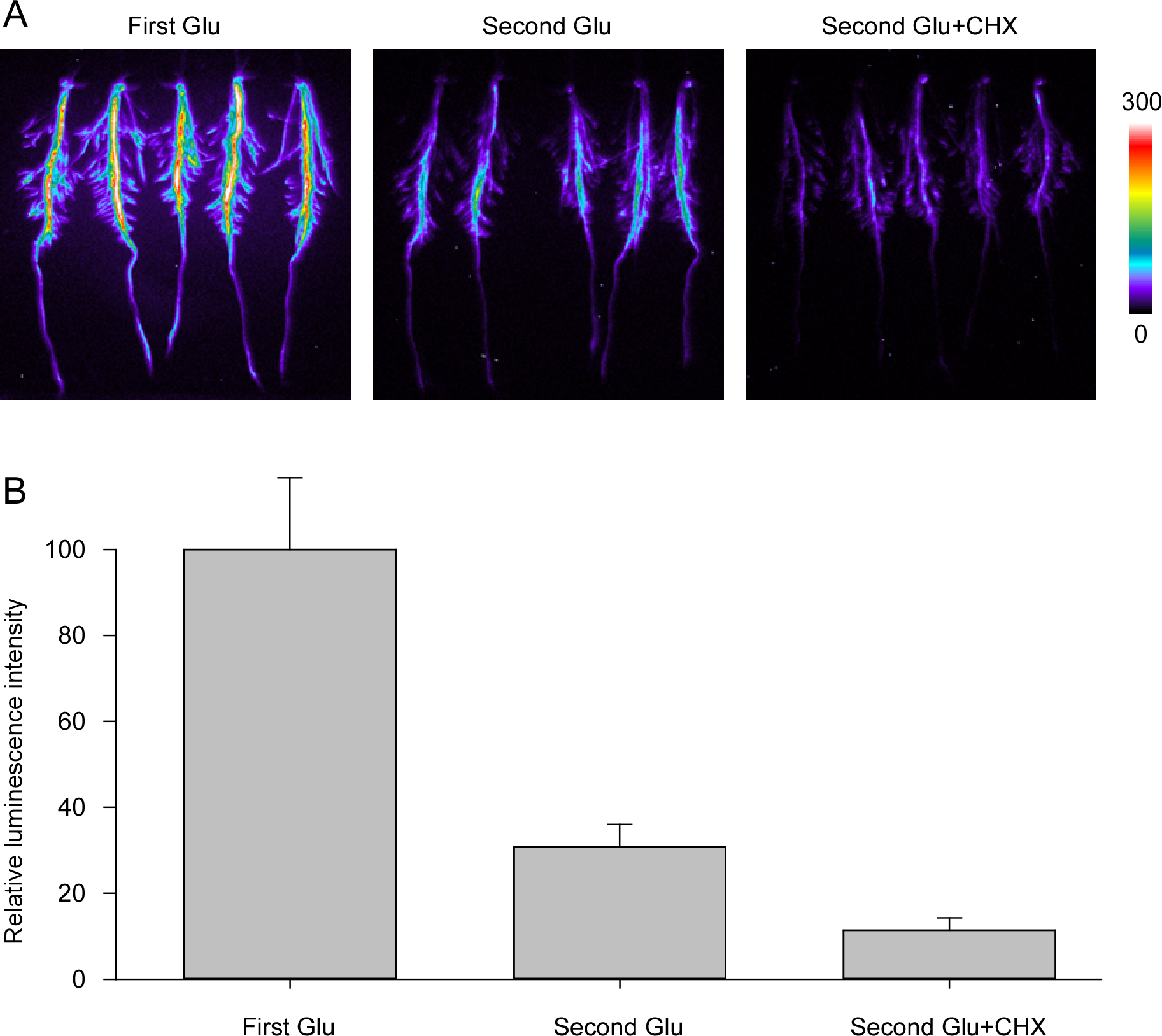

Supplement: Additional file 1: — Desensitization of Glu activated Ca2+ channels in rice roots. (A) Pseudocolor images of aequorin luminescence in response to two sequential Glu treatments. From left to right, the first application of Glu (left), the second application of Glu after1 hour’s recovery (middle) and second application of Glu pretreated with CHX (right). The relationship between luminescence intensity and the pseudocolor images are scaled by a pseudocolor bar and the numbers next to the pseudocolor bar are maximum and minimum values of luminescence intensity. (B) The bar chart of relative luminescence signal intensity of every treatment. The results were obtained from at least three independent experiments (mean ± sd; n = 10). (TIF 1537 kb) [file 12284_2016_81_MOESM1_ESM.tif]

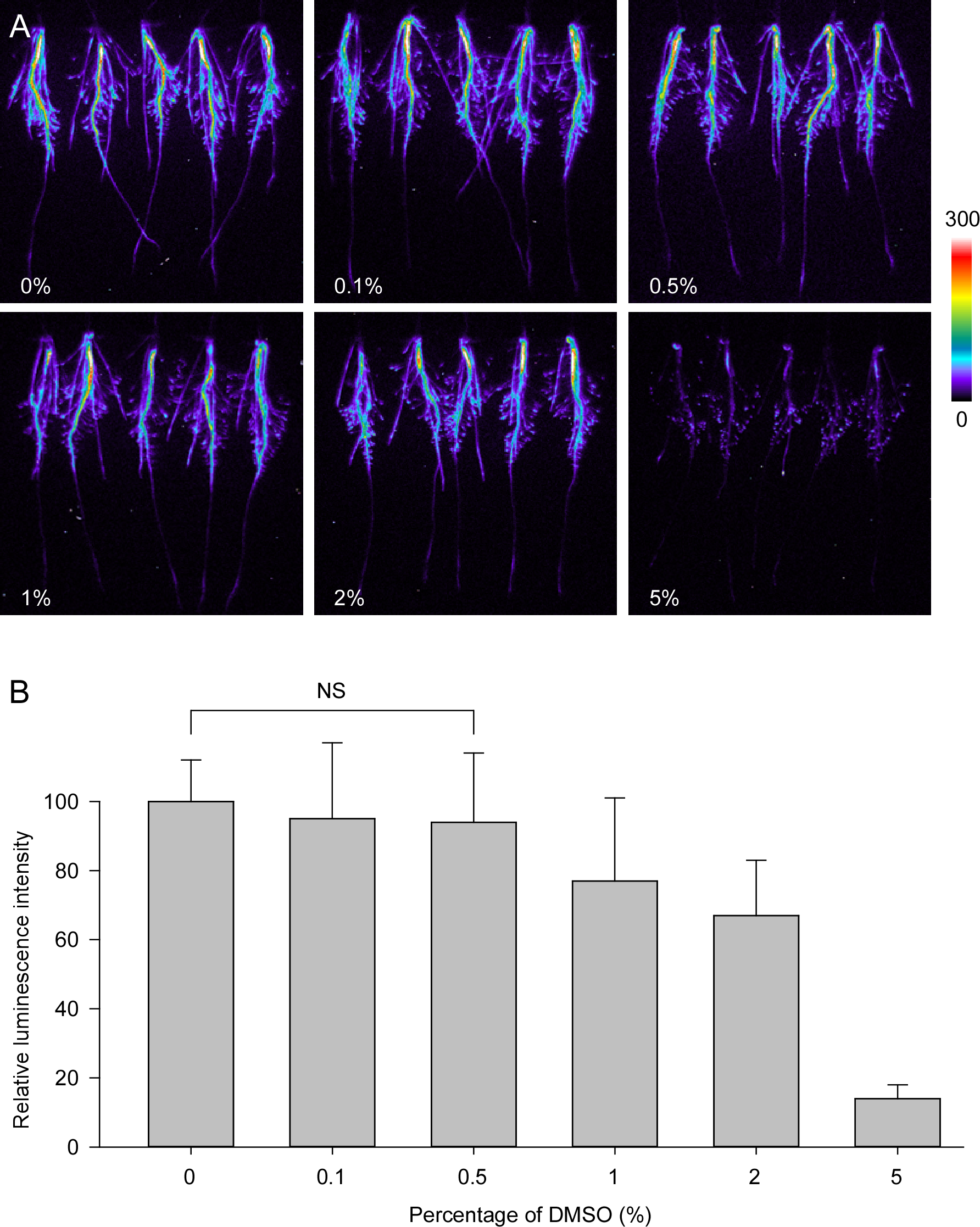

Supplement: Additional file 2: — The effect of DMSO in the Glu-induced [Ca2+]i increase. (A) Pseudocolor images of aequorin luminescence in roots treated by different percentages of DMSO. The relationship between luminescence intensity and the pseudocolor images are scaled by a pseudocolor bar and the numbers next to the pseudocolor bar are maximum and minimum values of luminescence intensity. (B) Relative luminescence signal intensity of every treatment. The results were obtained from at least three independent experiments (mean ± sd; n = 10; NS, not significant P > 0.05; Student’s t-test). (TIF 2858 kb) [file 12284_2016_81_MOESM2_ESM.tif]

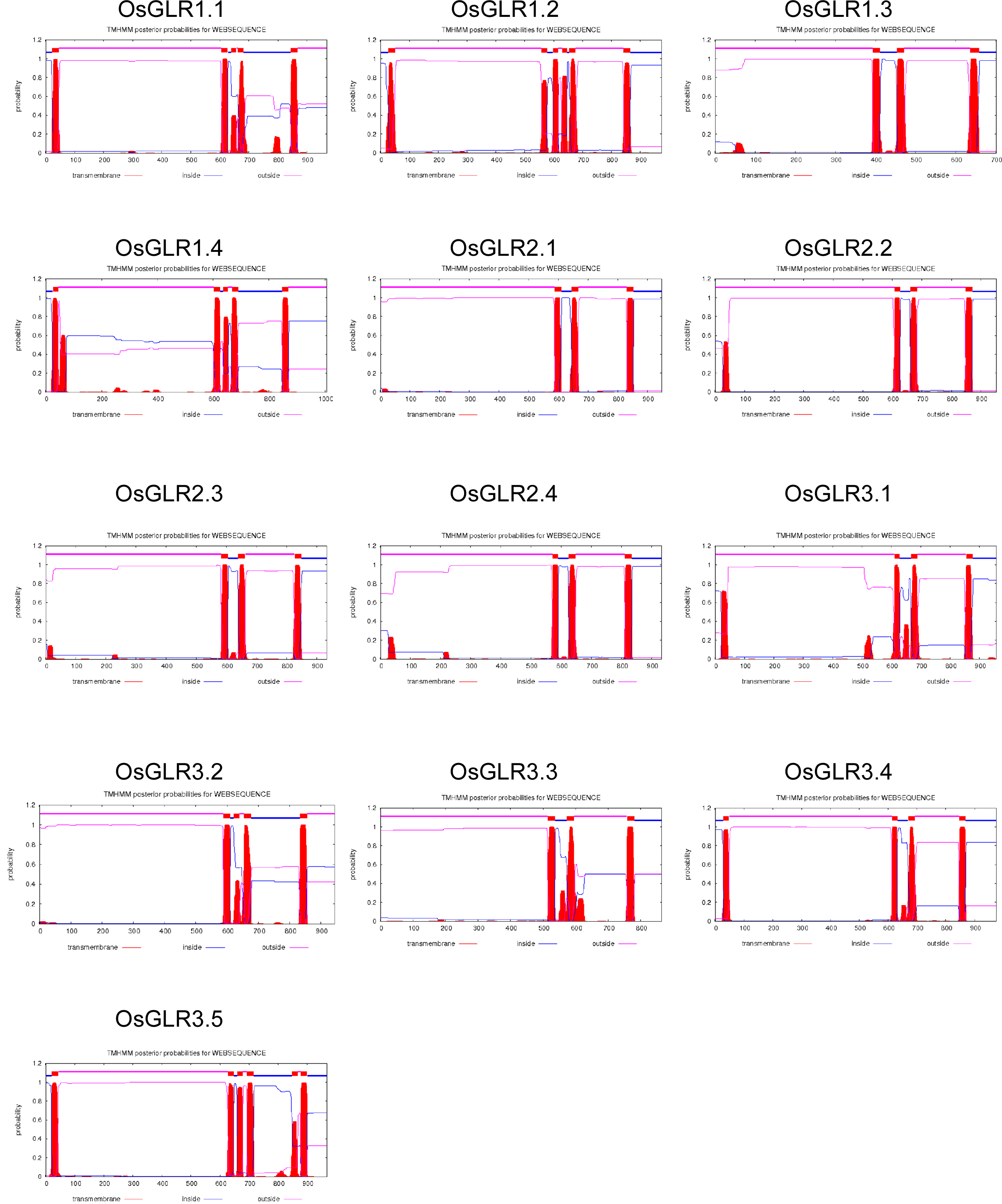

Supplement: Additional file 4: — Transmembrane helices structure predictions of OsGLR proteins. The transmembrane domains were estimated using TMHMM2: www.cbs.dtu.dk/services/TMHMM/. The red peaks showed the predicted transmembrane regions of proteins; the blue peaks showed the inside transmembrane domains; the pink peaks showed the outside transmembrane domain. (TIF 625 kb) [file 12284_2016_81_MOESM4_ESM.tif]

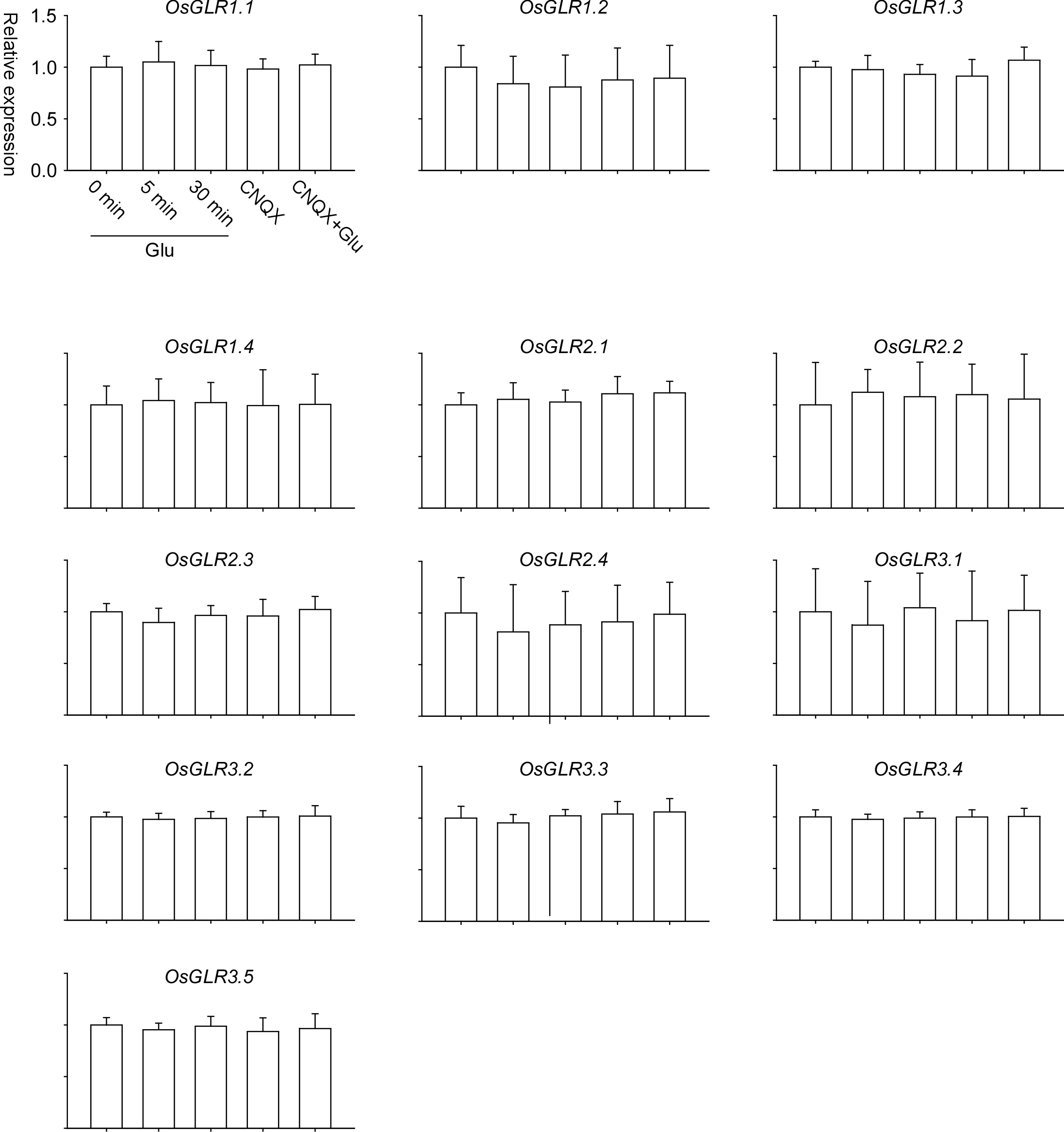

Supplement: Additional file 6: — qRT-PCR analysis of OsGLRs after treatment of Glu and CNQX. For Glu treatment, rice roots were treated with 20 mM Glu for 5 min or 30 min. For blocker treatment, rice roots were treated with 0.1 mM CNQX for 30 min. In addition, rice roots were also pretreated with 0.1 mM CNQX for 30 min, then treated with 20 mM Glu for 30 min (CNQX + Glu). Data for independent experiments are shown (mean ± sd; n = 3). (TIF 189 kb) [file 12284_2016_81_MOESM6_ESM.tif]

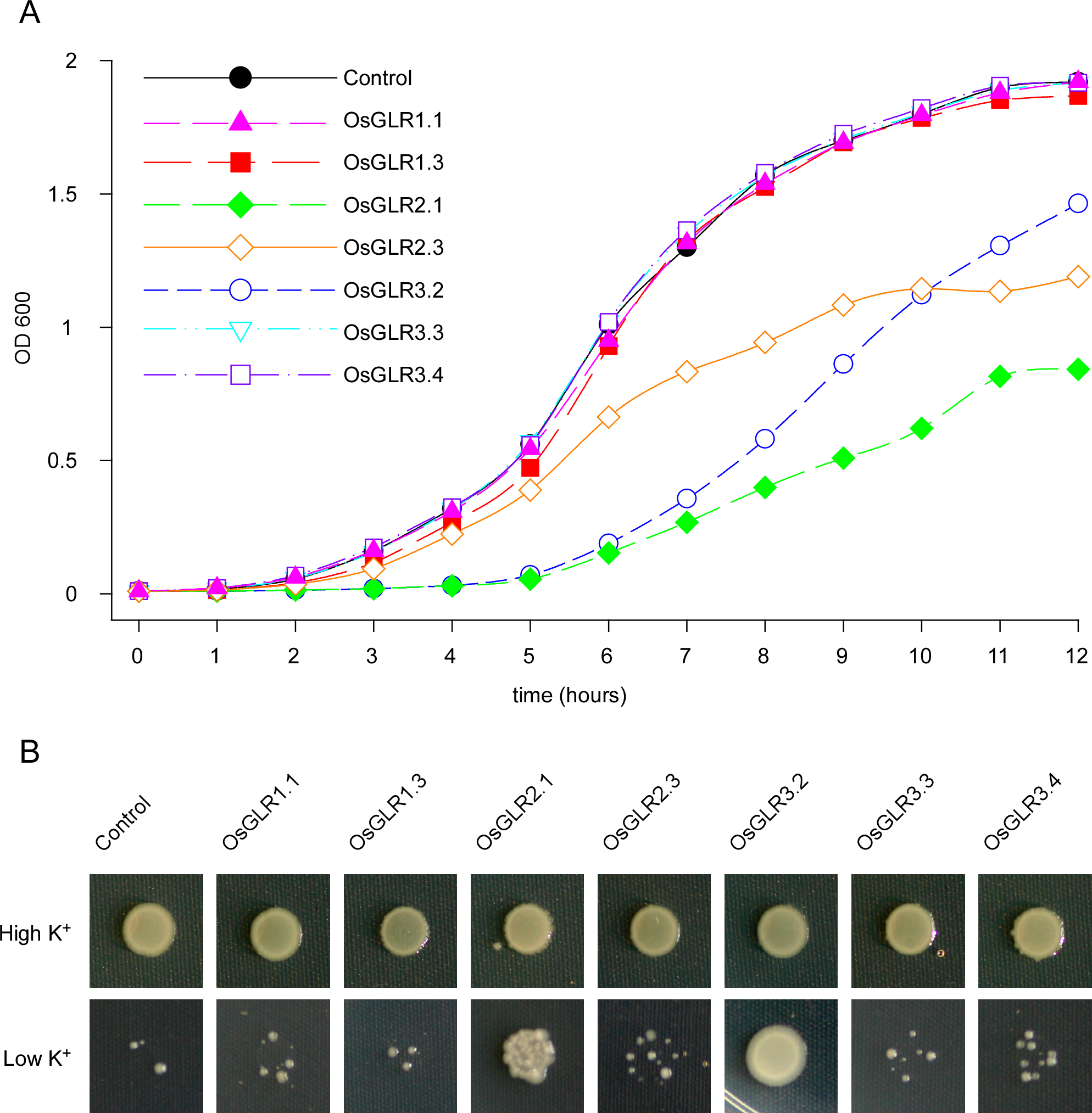

Supplement: Additional file 7: — OsGLRs mediate ion uptake in bacteria. (A) Bacterial growth rate of E. coli, transfected with different OsGLRs. Expression of OsGLR2.1, OsGLR2.3 and OsGLR3.2 reduced the growth rate of E. coli compared with the empty vector control. (B) K+ uptake-deficient E. coli mutant (strain LB650) transformed with different OsGLRs, was grown on solid selective medium with either high K+ (100 mM) or low K+ (2 mM). Expression of OsGLR2.1 and OsGLR3.2 enhance the growth of LB650 on low K+ medium compared with the empty vector control. (TIF 1891 kb) [file 12284_2016_81_MOESM7_ESM.tif]

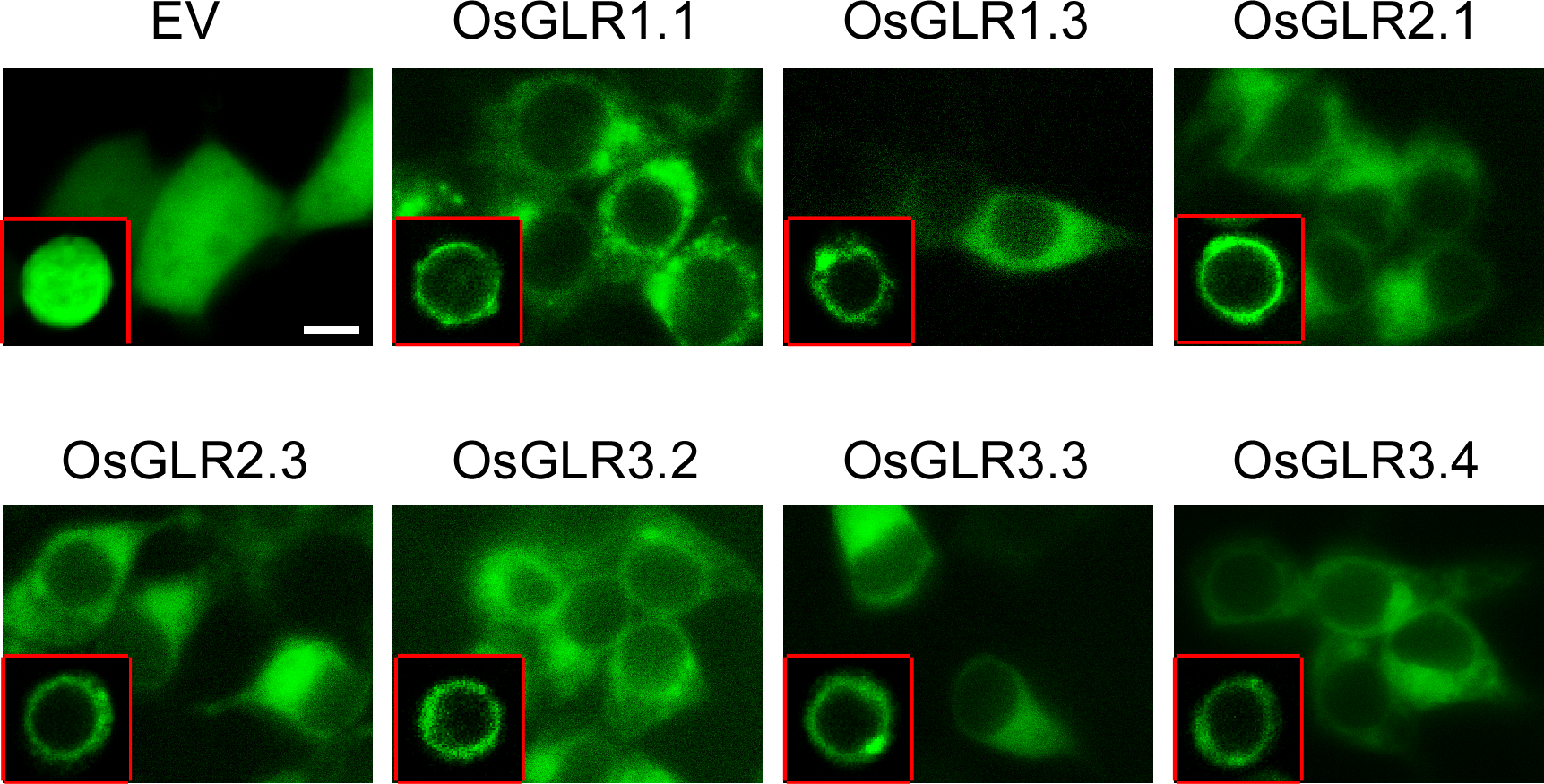

Supplement: Additional file 8: — Subcellular localization of OsGLRs in HEK293 cells. GFP fluorescence images of HEK293 cells expressing OsGLR-GFP. HEK cells treated with trypsin were inserted with red frames. EV, empty vector control. Bar = 10 μm. (TIF 1119 kb) [file 12284_2016_81_MOESM8_ESM.tif]

## Slide 1
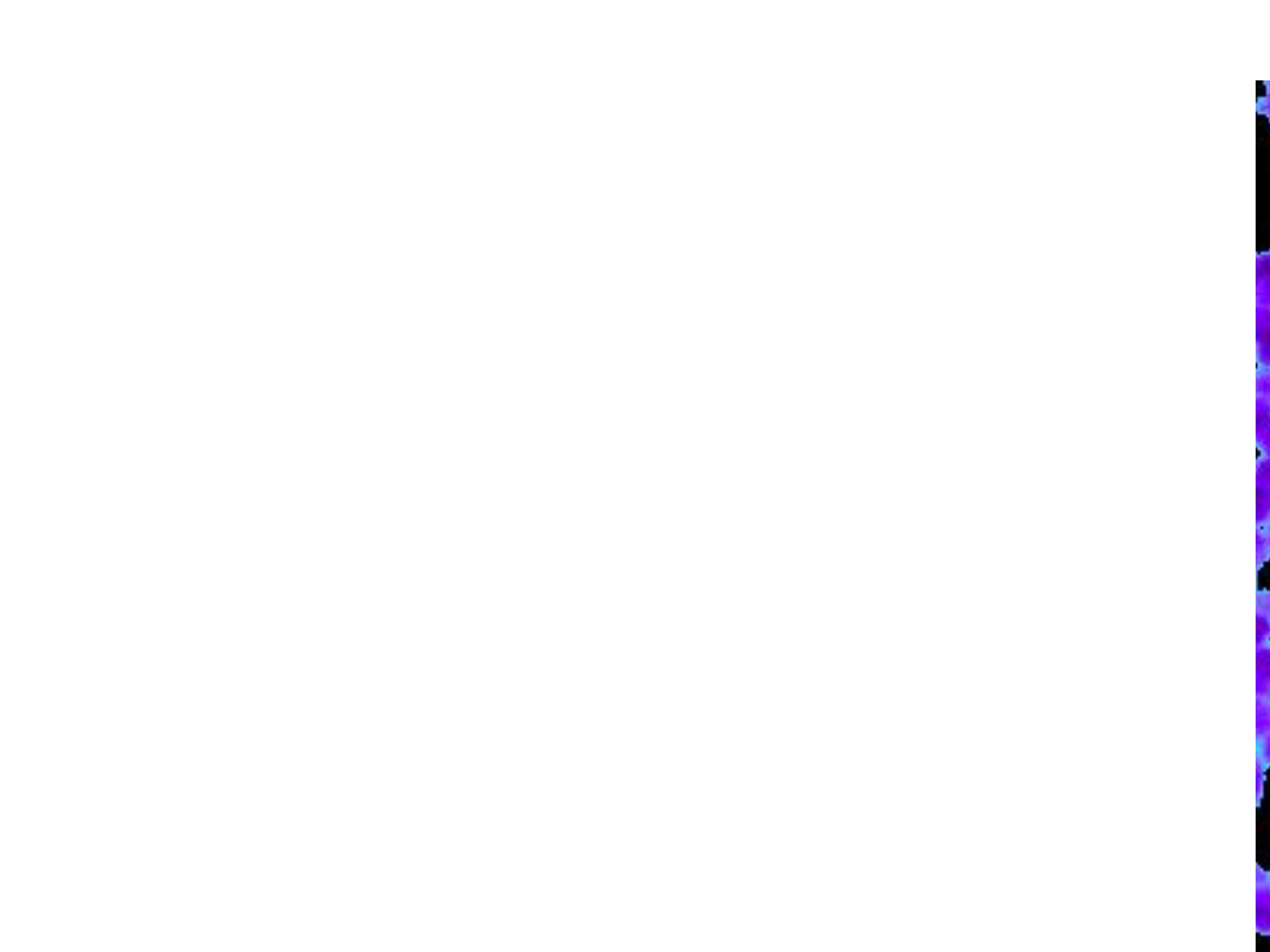

Supplement: Additional file 9: — The application of Glu triggered a [Ca2+]i increase in HEK cells expressing OsGLR2.1 (movie in the ppt file). (PPTX 19380 kb) [file 12284_2016_81_MOESM9_ESM.pptx]
