# Supplementary material for: Heterologous Expression and Functional Analysis of Rice GLUTAMATE RECEPTOR-LIKE Family Indicates its Role in Glutamate Triggered Calcium Flux in Rice Roots
Source: Rice (N Y). 2016 Mar 8;9:9. doi: 10.1186/s12284-016-0081-x (PMC4783324; doi:10.1186/s12284-016-0081-x)
Supplement: Additional file 5: — The sequence alignment of 20 AtGLRs and 13 OsGLRs (PDF 1229 kb) [file 12284_2016_81_MOESM5_ESM.pdf]

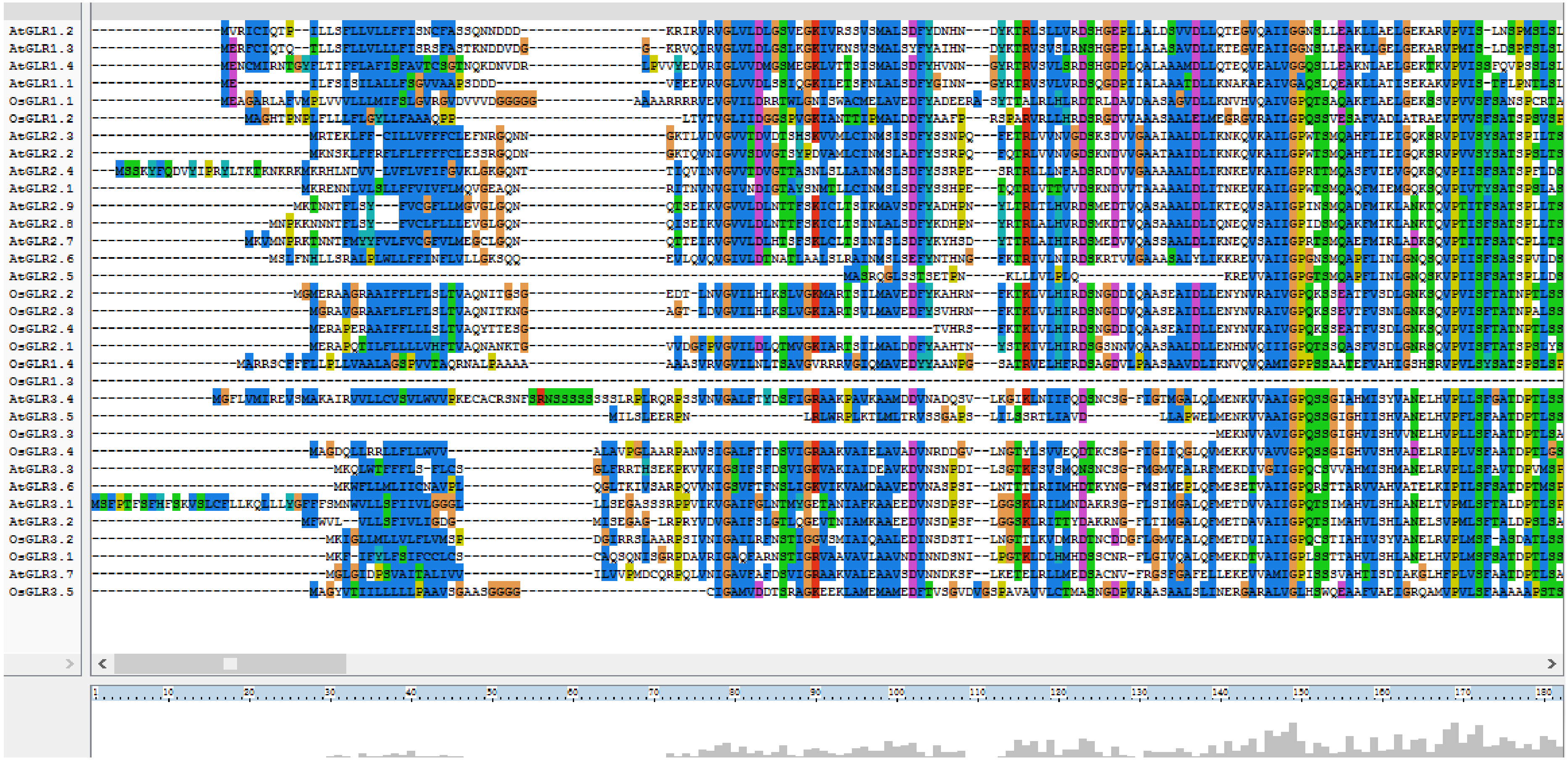
AtGLR1.2
AtGLR1.3
AtGLR1.4
AtGLR1.1
OsGLR1.1
OsGLR1.2
AtGLR2.3
AtGLR2.2
AtGLR2.4
AtGLR2.1
AtGLR2.9
AtGLR2.8
AtGLR2.7
AtGLR2.6
AtGLR2.5
OsGLR2.2
OsGLR2.3
OsGLR2.4
OsGLR2.1
OsGLR1.4
OsGLR1.3
AtGLR3.4
AtGLR3.5
OsGLR3.3
OsGLR3.4
AtGLR3.3
AtGLR3.6
AtGLR3.1
AtGLR3.2
OsGLR3.2
OsGLR3.1
AtGLR3.7
OsGLR3.5

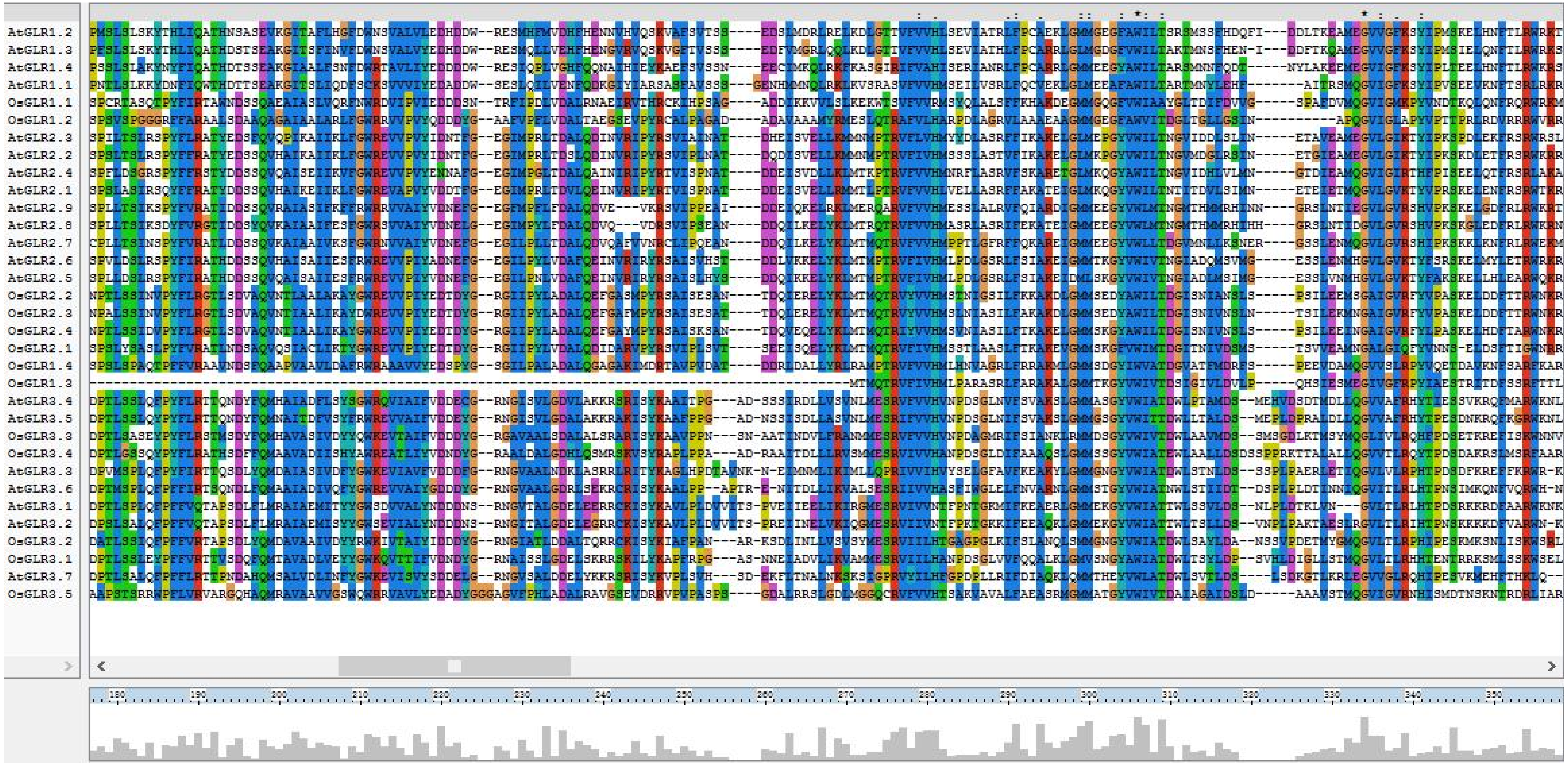
AtGLR1.2
AtGLR1.3
AtGLR1.4
AtGLR1.1
OsGLR1.1
OsGLR1.2
AtGLR2.3
AtGLR2.2
AtGLR2.4
AtGLR2.1
AtGLR2.9
AtGLR2.8
AtGLR2.7
AtGLR2.6
AtGLR2.5
OsGLR2.2
OsGLR2.3
OsGLR2.4
OsGLR2.1
OsGLR1.4
OsGLR1.3
AtGLR3.4
AtGLR3.5
OsGLR3.3
OsGLR3.4
AtGLR3.3
AtGLR3.6
AtGLR3.1
AtGLR3.2
OsGLR3.2
OsGLR3.1
AtGLR3.7
OsGLR3.5

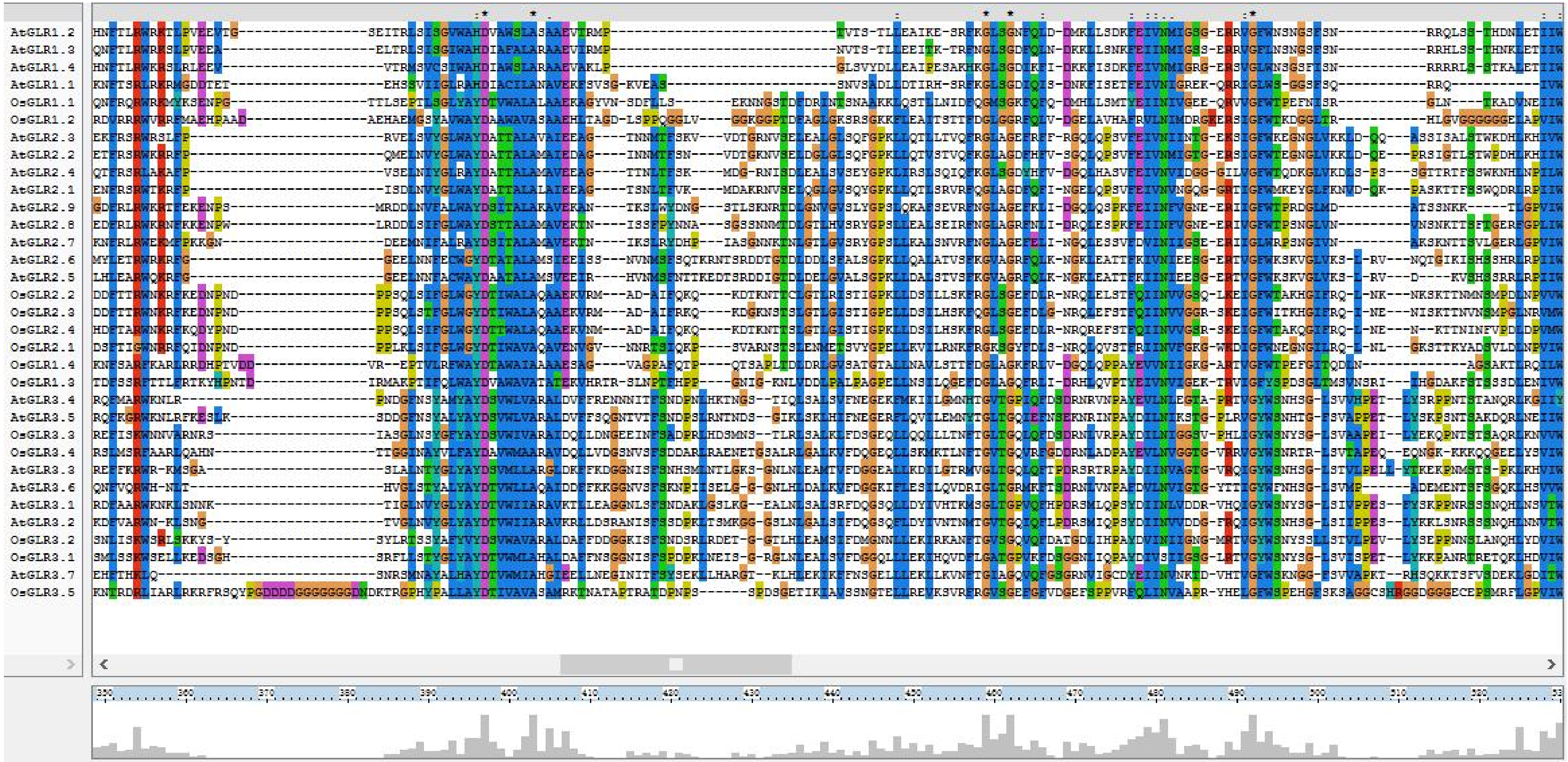
AtGLR1.2
AtGLR1.3
AtGLR1.4
AtGLR1.1
OsGLR1.1
OsGLR1.2
AtGLR2.3
AtGLR2.2
AtGLR2.4
AtGLR2.1
AtGLR2.9
AtGLR2.8
AtGLR2.7
AtGLR2.6
AtGLR2.5
OsGLR2.2
OsGLR2.3
OsGLR2.4
OsGLR2.1
OsGLR1.4
OsGLR1.3
AtGLR3.4
AtGLR3.5
OsGLR3.3
OsGLR3.4
AtGLR3.3
AtGLR3.6
AtGLR3.1
AtGLR3.2
OsGLR3.2
OsGLR3.1
AtGLR3.7
OsGLR3.5

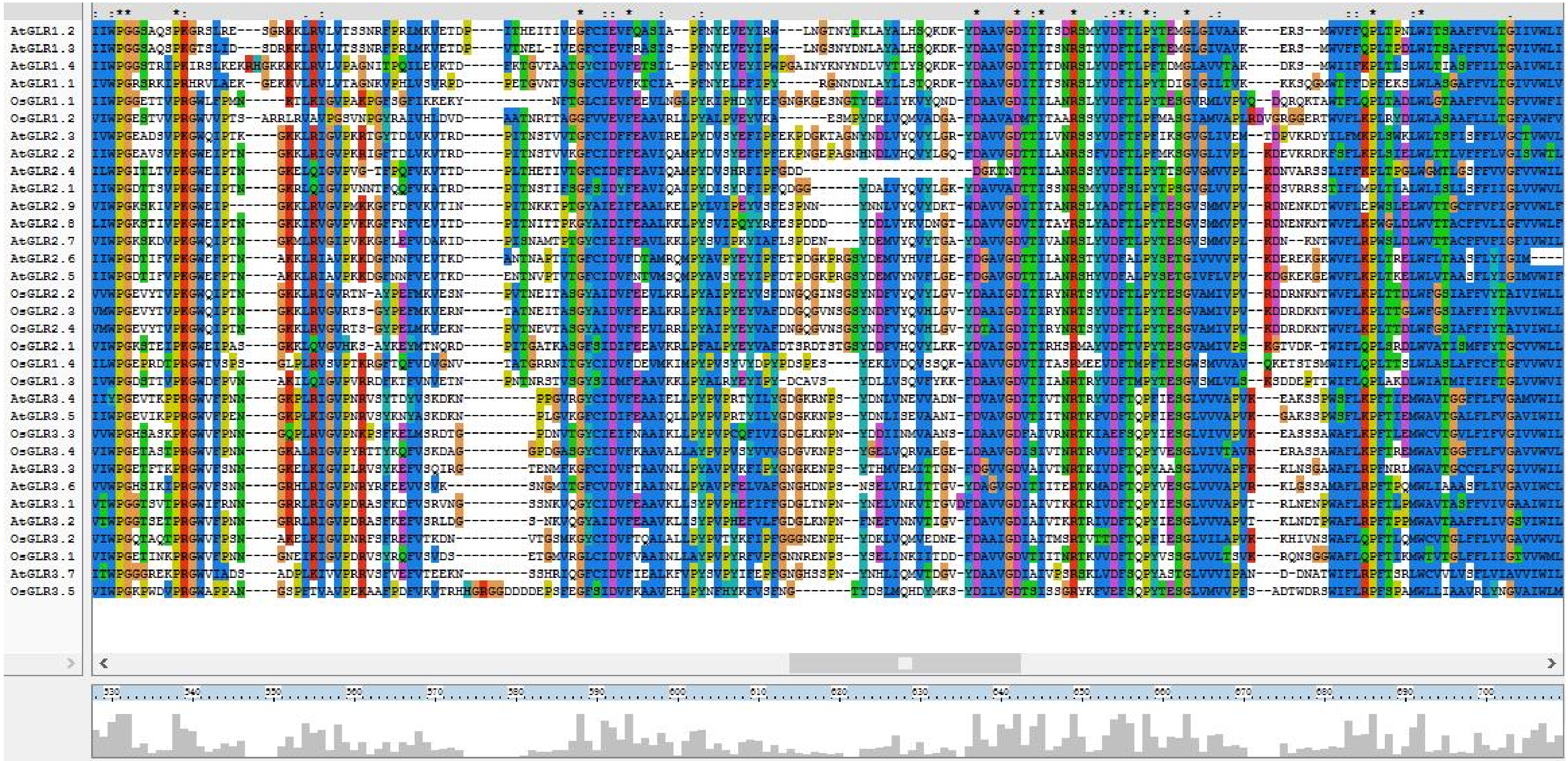
AtGLR1.2
AtGLR1.3
AtGLR1.4
AtGLR1.1
OsGLR1.1
OsGLR1.2
AtGLR2.3
AtGLR2.2
AtGLR2.4
AtGLR2.1
AtGLR2.9
AtGLR2.8
AtGLR2.7
AtGLR2.6
AtGLR2.5
OsGLR2.2
OsGLR2.3
OsGLR2.4
OsGLR2.1
OsGLR1.4
OsGLR1.3
AtGLR3.4
AtGLR3.5
OsGLR3.3
OsGLR3.4
AtGLR3.3
AtGLR3.6
AtGLR3.1
AtGLR3.2
OsGLR3.2
OsGLR3.1
AtGLR3.7
OsGLR3.5
530
540
550
560
570
580
590
600
610
620
630
640
650
660
670
680
690
700

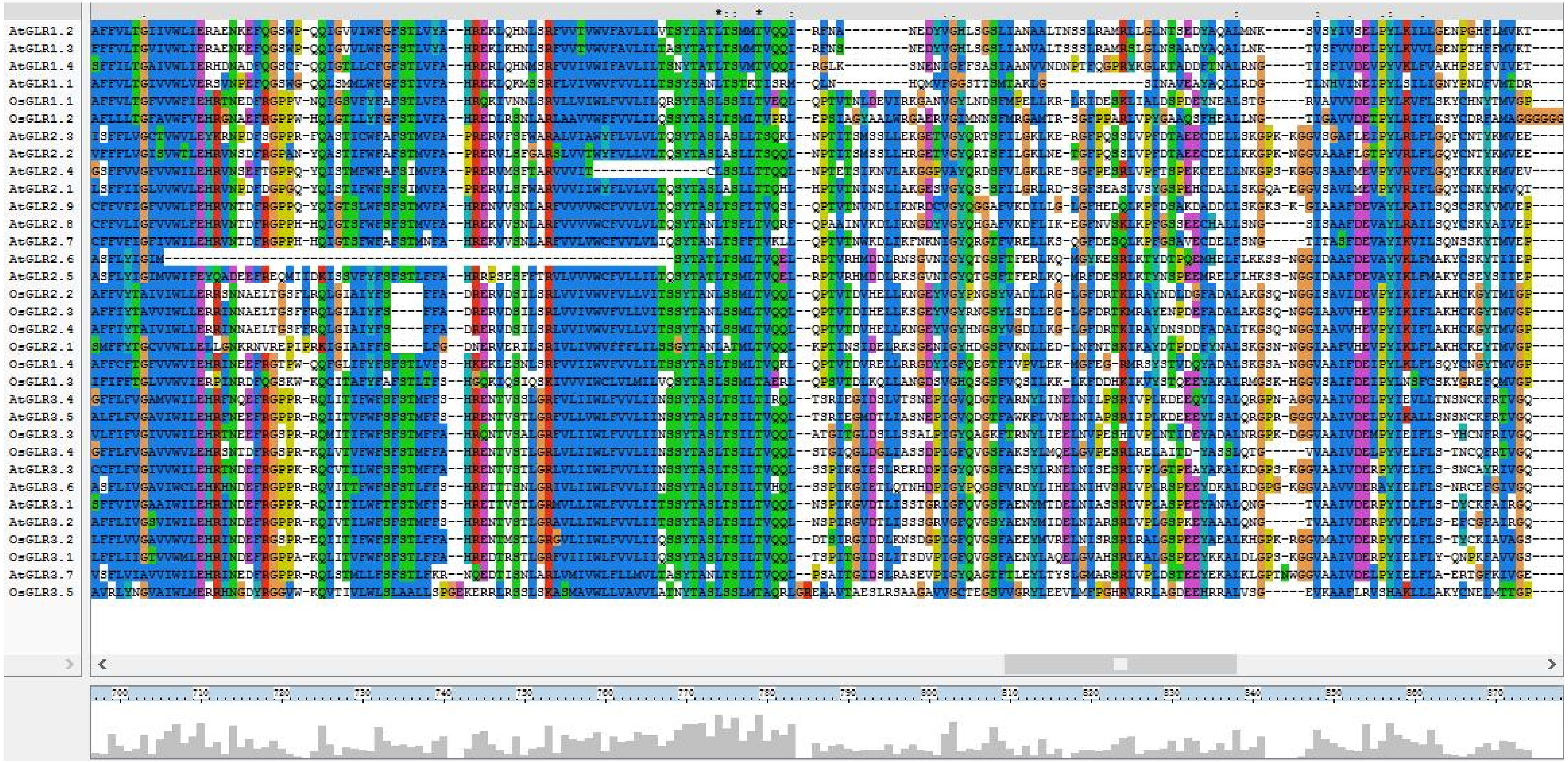
AtGLR1.2
AtGLR1.3
AtGLR1.4
AtGLR1.1
OsGLR1.1
OsGLR1.2
AtGLR2.3
AtGLR2.2
AtGLR2.4
AtGLR2.1
AtGLR2.9
AtGLR2.8
AtGLR2.7
AtGLR2.6
AtGLR2.5
OsGLR2.2
OsGLR2.3
OsGLR2.4
OsGLR2.1
OsGLR1.4
OsGLR1.3
AtGLR3.4
AtGLR3.5
OsGLR3.3
OsGLR3.4
AtGLR3.3
AtGLR3.6
AtGLR3.1
AtGLR3.2
OsGLR3.2
OsGLR3.1
AtGLR3.7
OsGLR3.5
700
710
720
730
740
750
760
770
780
790
800
810
820
830
840
850
860
870

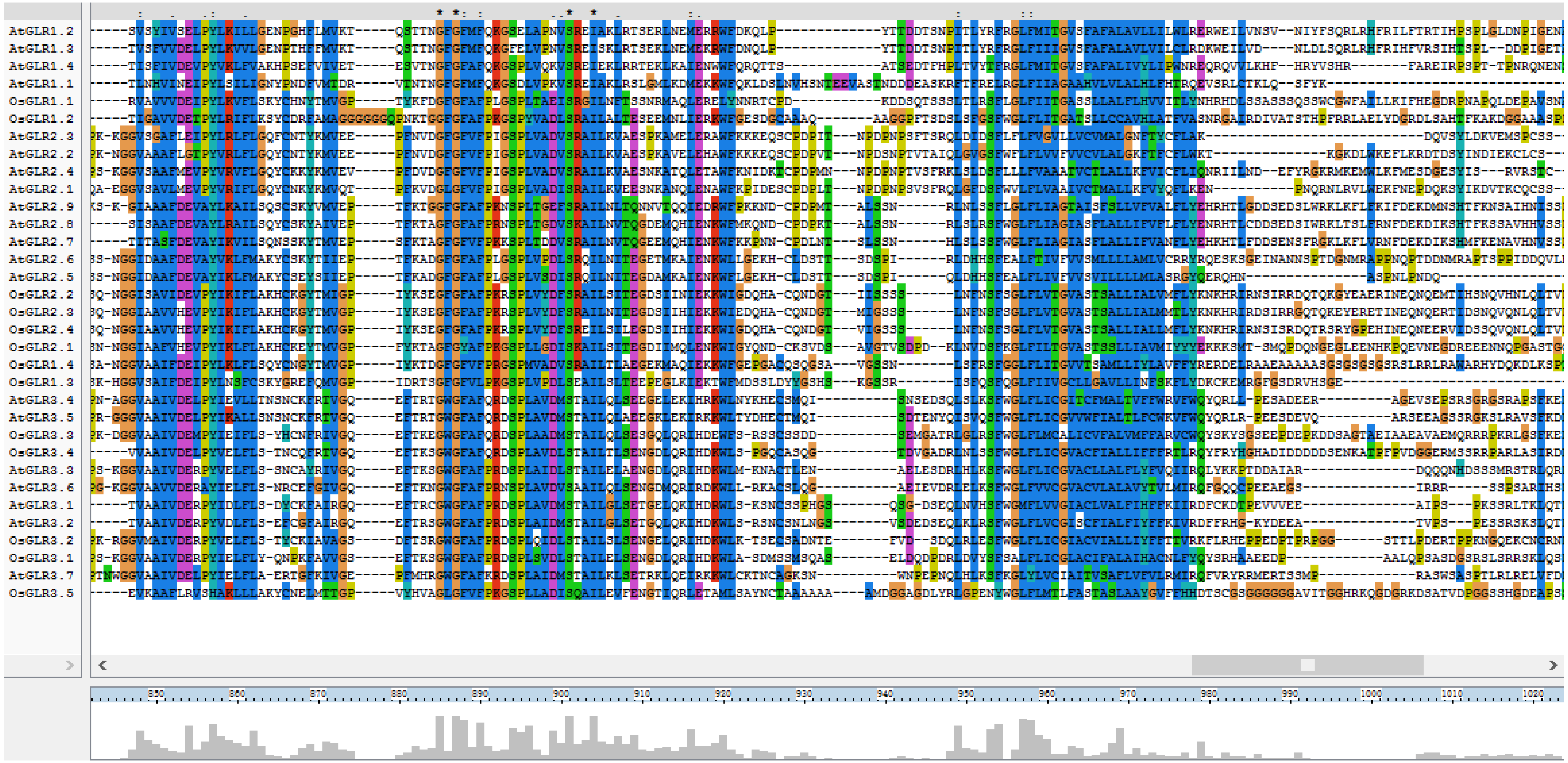
AtGLR1.2
AtGLR1.3
AtGLR1.4
AtGLR1.1
OsGLR1.1
OsGLR1.2
AtGLR2.3
AtGLR2.2
AtGLR2.4
AtGLR2.1
AtGLR2.9
AtGLR2.8
AtGLR2.7
AtGLR2.6
AtGLR2.5
OsGLR2.2
OsGLR2.3
OsGLR2.4
OsGLR2.1
OsGLR1.4
OsGLR1.3
AtGLR3.4
AtGLR3.5
OsGLR3.3
OsGLR3.4
AtGLR3.3
AtGLR3.6
AtGLR3.1
AtGLR3.2
OsGLR3.2
OsGLR3.1
AtGLR3.7
OsGLR3.5

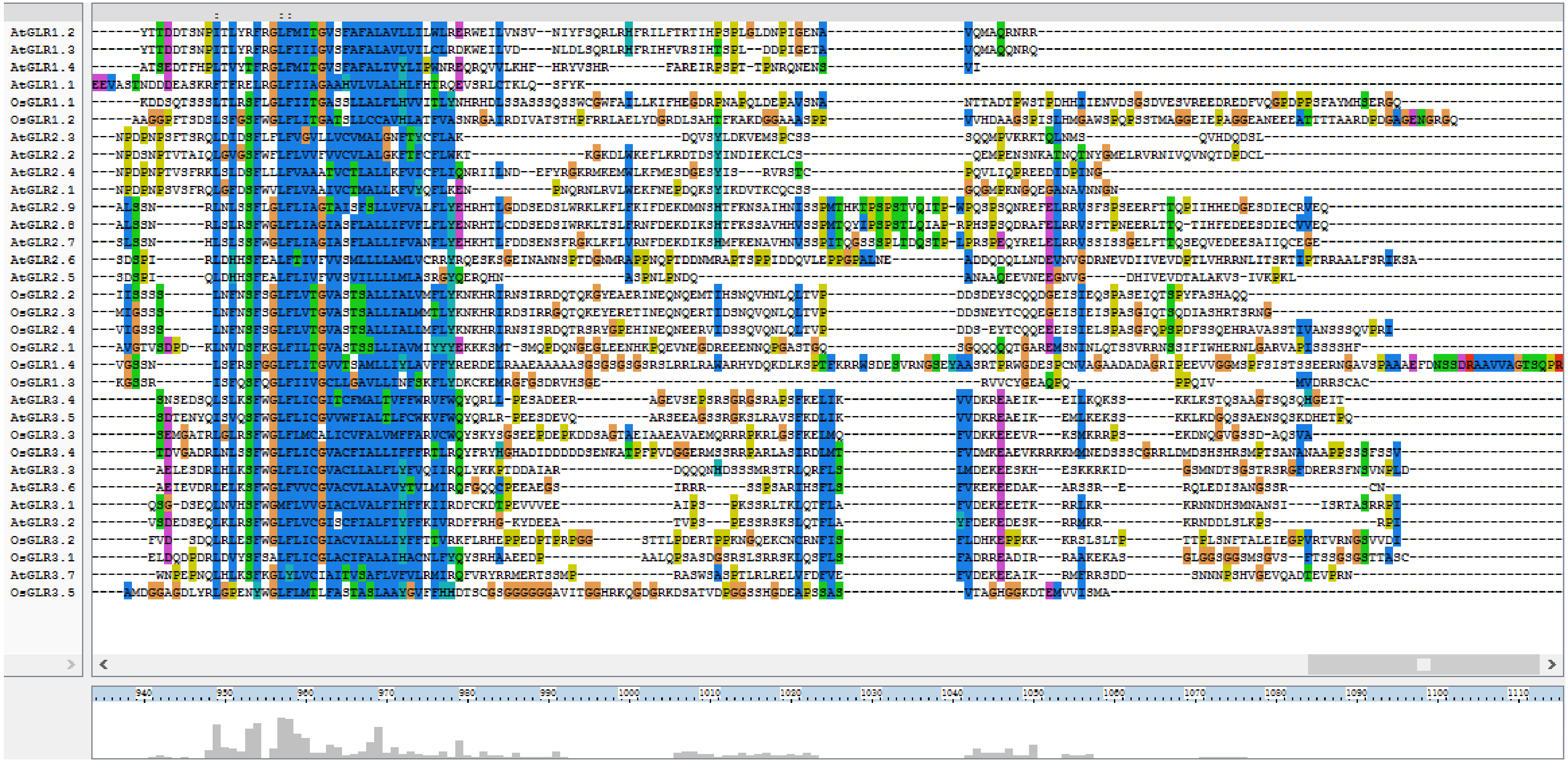
AtGLR1.2
AtGLR1.3
AtGLR1.4
AtGLR1.1
OsGLR1.1
OsGLR1.2
AtGLR2.3
AtGLR2.2
AtGLR2.4
AtGLR2.1
AtGLR2.9
AtGLR2.8
AtGLR2.7
AtGLR2.6
AtGLR2.5
OsGLR2.2
OsGLR2.3
OsGLR2.4
OsGLR2.1
OsGLR1.4
OsGLR1.3
AtGLR3.4
AtGLR3.5
OsGLR3.3
OsGLR3.4
AtGLR3.3
AtGLR3.6
AtGLR3.1
AtGLR3.2
OsGLR3.2
OsGLR3.1
AtGLR3.7
OsGLR3.5
940
950
960
970
980
990
1000
1010
1020
1030
1040
1050
1060
1070
1080
1090
1100
1110
